# Association of Epigenetic Age Acceleration and Mitochondrial DNA‐Based Aging Metrics Provides Insights Into Mechanisms of Aging‐Related Diseases

**DOI:** 10.1111/acel.70279

**Published:** 2025-10-24

**Authors:** Mengyao Wang, Yinan Zheng, Meng Lai, Emmanuel Saake, Xue Liu, Xiuqing Guo, Kent D. Taylor, Tianxiao Huan, Roby Joehanes, Drew R. Nannini, Kai Zhang, Nicole J. Lake, Christina A. Castellani, Stephen S. Rich, Jerome I. Rotter, Yongmei Liu, Laura M. Raffield, April P. Carson, Myriam Fornage, Jiantao Ma, Dan E. Arking, Lifang Hou, Daniel Levy, Chunyu Liu

**Affiliations:** ^1^ Department of Biostatistics Boston University School of Public Health Boston Massachusetts USA; ^2^ Department of Preventive Medicine Northwestern University Feinberg School of Medicine Chicago Illinois USA; ^3^ Faculty of Computing & Data Sciences Boston University Boston Massachusetts USA; ^4^ Bioinformatics Program, Faculty of Computing and Data Science Boston University Boston Massachusetts USA; ^5^ The Institute for Translational Genomics and Population Sciences, Department of Pediatrics, the Lundquist Institute for Biomedical Innovation Harbor‐UCLA Medical Center Torrance California USA; ^6^ Population Sciences Branch, National Heart, Lung, and Blood Institute (NHLBI) National Institutes of Health Bethesda Maryland USA; ^7^ Department of Environmental Health Sciences, College of Integrated Health Sciences University at Albany, State University of New York Rensselaer New York USA; ^8^ Department of Genetics Yale School of Medicine New Haven Connecticut USA; ^9^ Departments of Pathology and Laboratory Medicine & Epidemiology and Biostatistics Western University London Ontario Canada; ^10^ McKusick‐Nathans Institute, Department of Genetic Medicine Johns Hopkins University School of Medicine Baltimore Maryland USA; ^11^ Department of Genome Sciences University of Virginia Charlottesville Virginia USA; ^12^ Divisions of Cardiology and Neurology, Department of Medicine Duke University Medical Center Durham North Carolina USA; ^13^ Department of Genetics University of North Carolina at Chapel Hill Chapel Hill North Carolina USA; ^14^ Department of Medicine University of Mississippi Medical Center Jackson Mississippi USA; ^15^ Human Genetics Center, Department of Epidemiology, School of Public Health The University of Texas Health Science Center at Houston Houston Texas USA; ^16^ Brown Foundation Institute of Molecular Medicine, McGovern Medical School The University of Texas Health Science Center at Houston Houston Texas USA; ^17^ Nutrition Epidemiology and Data Science, Friedman School of Nutrition Science and Policy Tufts University Boston Massachusetts USA; ^18^ Framingham Heart Study, National Heart, Lung, and Blood Institute (NHLBI) Framingham Massachusetts USA

**Keywords:** DNA methylation, epigenetic aging, heteroplasmy, mitochondrial DNA

## Abstract

Investigating the interplay between mitochondrial DNA (mtDNA) variations and epigenetic aging metrics may elucidate biological mechanisms associated with age‐related diseases. We estimated epigenetic age acceleration (EAA) metrics from DNA methylation data and derived mtDNA metrics, including heteroplasmic variants and mtDNA copy number (mtDNA CN) from whole genome sequencing. Linear regressions and meta‐analyses were conducted to assess associations between EAA and mtDNA metrics, adjusting for chronological age, self‐identified sex, and other covariates in 6,316 participants (58% female, 41% non‐White Americans). Mediation analysis was conducted to examine whether EAA mediated the relationship between mtDNA CN and metabolic traits. A higher burden of rare heteroplasmic variants was associated with accelerations of first‐generation EAA metrics, while a lower level mtDNA CN was associated with accelerations of second‐ and third‐generation EAA metrics. For example, one standard deviation (SD) higher MSS, a score based on the predicted functions of rare heteroplasmic variants, was associated with a 0.22‐year higher EAA by the Hannum method (*p* = 1.3E‐6) among all participants, while one SD lower mtDNA CN was associated with higher DunedinPACE (*β* = −0.005, *p* = 6.0E‐4). No significant association was observed between the heteroplasmy burden of common variants and EAAs. Furthermore, we observed DunedinPACE mediated 11.1% and 10.8% of the associations of mtDNA CN with obesity and T2DM in older FHS participants, respectively. Our analysis indicated that higher levels of heteroplasmy burden of rare variants and lower mtDNA CN were associated with accelerated epigenetic aging, and these associations showed stronger magnitudes among older participants.

## Graphical abstract

Older participants exhibit lower mtDNA copy number (CN) and a higher rare heteroplasmy burden, while common heteroplasmy burden remains relatively stable. Higher epigenetic age acceleration is associated with lower mtDNA CN and higher rare heteroplasmy burden in 6316 participants.

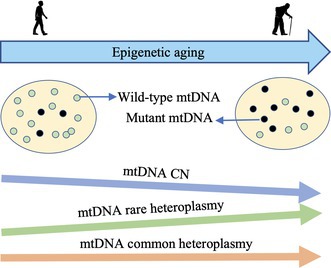

## 1 Introduction

Aging is a gradual decline in physiologic function, resulting in greater risks of disease and mortality (Kyriazis 2020). However, biological aging can vary among people of the same chronological age. Nine aging‐related cellular and molecular hallmarks, including epigenetic alterations and mitochondrial dysfunction, have been proposed as contributing factors to age‐related diseases (López‐Otín et al. 2013). Alterations in epigenetic regulation have been associated with disease progression, where DNA methylation (DNAm) has been the most extensively studied. DNAm involves the addition of a methyl group to the carbon 5 position of cytosine (Tost 2010). Alterations in DNAm are associated with aging and age‐related diseases, providing the basis for estimating biological age (Fransquet et al. 2019; Horvath 2013; Hannum et al. 2013). Epigenetic age acceleration (EAA) is defined as the difference between DNAm‐based biological age and chronological age. A higher EAA indicates that an individual's biological aging is occurring faster than expected for their chronological age. Numerous studies have shown that higher EAA is a strong predictor of shorter lifespan and is associated with elevated risk of mortality, frailty, and a wide range of age‐related diseases, including cardiovascular disease (CVD), neurodegenerative disorders, and cancer (Horvath et al. 2016; Levine et al. 2018; Lu et al. 2019; Higgins‐Chen et al. 2022; Sun et al. 2024; Seligman et al. 2022; Jain et al. 2022; Perna et al. 2016).

Mitochondria are important organelles for regulating cellular activities beyond producing cellular energy (Wallace 2012). The maternally inherited human mitochondrial genome (mtDNA) consists of 16,569 base pairs, encoding 37 genes (Voet et al. 2016). Hundreds to thousands of mtDNA molecules exist in one cell, resulting in two metrics, mtDNA copy number (mtDNA CN) and heteroplasmy. Previous studies have found lower mtDNA CN was associated with older age, all‐cause mortality (Ashar et al. 2015), poorer general health (Mengel‐From et al. 2014), lower cognitive function (Wei et al. 2017; Gui et al. 2015), and CVD (Zhang et al. 2017; Ashar et al. 2017; Liu, Longchamps, et al. 2021). Heteroplasmy is a phenomenon where two or more nucleotides co‐exist at the same mtDNA locus in the same individual (Stewart and Chinnery 2015). Higher heteroplasmy burden has been associated with older age (Ding et al. 2015; Liu, Fetterman, et al. 2021) and may contribute to age‐related diseases (Stewart and Chinnery 2015; Wallace 2013; Hong et al. 2023). As major hallmarks of aging, alterations in both epigenetic regulation and mtDNA metrics reflect dynamic changes. While several studies have explored the potential regulatory role of mtDNA homoplasmic (Bellizzi et al. 2012; Vivian et al. 2017; Lee et al. 2017) and heteroplasmic variants (Kopinski et al. 2019) on the nuclear epigenome, the relationship between these two hallmarks of aging remains to be elucidated. Understanding how these hallmarks interact may offer valuable insights into the biological mechanisms underlying age‐related diseases (Lopes 2020).

We hypothesized that mtDNA CN and heteroplasmy burden scores derived from rare and common variants capture distinct dimensions of mitochondrial biology and would show differential associations with biological aging and metabolic traits. Rare variants may reflect recent, potentially deleterious mutational events associated with pathological aging, whereas common variants may represent stable, inherited variation. In contrast, mtDNA CN serves as a broader indicator of mitochondrial abundance and functional capacity. To test the hypothesis, we conducted association analyses between these mtDNA metrics and several EAA metrics in 6316 participants from four cohort studies. In particular, we compared heteroplasmy burden derived from common and rare heteroplasmic variants in relation to EAAs to investigate their distinct roles in the biological aging process. We then evaluated the relationship among mtDNA CN, epigenetic aging metrics, and metabolic traits such as obesity and type 2 diabetes mellitus (T2DM), using both mediation and Mendelian randomization analyses. Examining the relationship between these two molecular aging metrics may reveal insights into their role in age‐related diseases and may guide therapeutic strategies to slow the aging or mitigate its effects.

## 2 Methods

### 2.1 Study Participants

This study included four longitudinal cohort studies (Table 1, Supplemental Methods): Coronary Artery Risk Development in Young Adults (CARDIA; *n* = 2289) (Lloyd‐Jones et al. 2021), Framingham Heart Study (FHS; *n* = 1745) (Dawber et al. 1951; Andersson et al. 2019; Feinleib et al. 1975; Splansky et al. 2007), Jackson Heart Study (JHS; *n* = 1424) (Fuqua et al. 2005; Carpenter et al. 2004), and Multi‐Ethnic Study of Atherosclerosis (MESA; *n* = 858) (Blaha and DeFilippis 2021). FHS, JHS, and MESA consisted primarily of middle‐aged and older participants (mean age: 55–61 years), while CARDIA included younger participants (mean age: 46 years). Self‐reported race/ethnicity and self‐identified sex (i.e., males and females) were used in all statistical analyses. FHS consisted of self‐reported White American participants, whereas JHS included self‐reported Black American participants. CARDIA included participants from both ethnic groups and MESA consisted of Hispanic Americans in addition to Black American and White American participants. In MESA, we further grouped self‐reported Hispanic American participants with White American participants in the statistical analysis (with an index variable for ethnicity) because of the similarity of the genetic effects between these two ethnic groups (Mogil et al. 2018). Protocols for participant examinations and genetic material collection were approved by the Institutional Review Boards at the respective research sites. All participants provided written, informed consent for genetic studies, and all research was performed in accordance with relevant guidelines and regulations.

**TABLE 1:** Participant characteristics.

	FHS (*n* = 1745)	JHS (*n* = 1424)	MESA (*n* = 858)	CARDIA (*n* = 2289)
Race/Ethnicity (%)				
WA	1745 (100%)	0 (0%)	678 (79.0%)	1314 (57.4%)
BA	0 (0%)	1424 (100%)	180 (21.0%)	975 (42.6%)
Age	60.8 (13.6)	55.3 (12.4)	60.2 (9.7)	45.6 (6.3)
Female	951 (54.5%)	887 (62.3%)	460 (53.6%)	1372 (59.9%)
MH_com_count > 0	1234 (70.7%)	833 (58.5%)	589 (68.6%)	1414 (61.8%)
MHcount > 0	550 (31.5%)	399 (28.0%)	244 (28.4%)	565 (24.7%)
MSS > 0.25	277 (15.9%)	149 (10.5%)	87 (10.1%)	230 (10.0%)
Standardized mtDNA CN	0 (1)	0 (1)	0 (1)	0 (1)

*Note:* Continuous variables are presented as mean ± standard deviation, and categorical variables are presented as frequency and proportion.

Abbreviations: BA, Black American participants; CARDIA, Coronary Artery Risk Development in Young Adults Study; FHS, Framingham Heart Study; JHS, Jackson Heart Study; MESA, Multi‐Ethnic Study of Atherosclerosis; MH_com_count, mitochondrial common heteroplasmic variants count; MHcount, mitochondrial rare heteroplasmic variants count; MSS, mitochondrial local constraint score sum based on rare variants; mtDNA CN, mitochondrial DNA copy number; WA, White American participants.

### 2.2 DNA Methylation Measurements

Whole blood samples were routinely collected from study participants at health exams across cohorts and underwent DNA extraction. Genomic DNA was assayed with either the Infinium HumanMethylation450 BeadChip array (in FHS and MESA) or the Infinium MethylationEPIC BeadChip array (in CARDIA and JHS) (Table S1, Supporting Methods). Bisulfite conversion was performed, followed by whole genome amplification, fragmentation, array hybridization, and single‐base pair extension based on the manufacturer's protocols (Bibikova et al. 2009). Methylation *β*‐values were calculated with measured intensities of methylated and unmethylated probes. In all cohorts, we excluded low‐quality probes including probes with a high missing rate (> 20%), a detection *p*‐value > 0.01, or single nucleotide polymorphisms (SNPs) at CpG sites or ≤ 10 bp of single base extension (Chen et al. 2013; Kuan et al. 2010). We further excluded low‐quality samples including samples with a missing rate > 1%, with outliers according to multi‐dimensional scaling (MDS) analysis (Taguchi and Oono 2005), or poorly matching to the 68 SNP genotypes on the Infinium HumanMethylation 450K and MethylationEPIC BeadChip array.

### 2.3 Estimation of DNA Methylation Aging Metrics

The first‐generation DNAm clocks comprise Horvath age (Horvath 2013) and Hannum age (Hannum et al. 2013), while second‐generation clocks include PhenoAge (Levine et al. 2018) and GrimAge (Lu et al. 2019). Principal component (PC)‐based versions of these clocks have been developed to reduce unobserved technical noise (Higgins‐Chen et al. 2022). We calculated the PC‐based clocks, referred to as PC‐Horvath, PC‐Hannum, PC‐PhenoAge, and PC‐GrimAge. Briefly, PCs were estimated for each epigenetic clock, with all CpG probes passing quality control. Elastic net modeling was utilized to train PC‐based clocks with 10‐fold cross‐validation (Higgins‐Chen et al. 2022). We calculated cohort‐ and race/ethnicity‐specific, as well as lab‐specific (in FHS), PC‐based clocks. Epigenetic age acceleration (EAA) metrics, EAAHorvath, EAAHannum, EAAPheno, and EAAGrim, were defined as residuals from regressing each PC‐based clock on chronological age within cohort and race/ethnicity strata. Unlike earlier clocks, the third‐generation epigenetic clock, DunedinPACE, estimates the yearly pace of aging instead of age in years. We calculated DunedinPACE in cohort‐ and race/ethnicity‐specific groups, as well as in lab‐specific groups (in FHS).

### 2.4 Whole Genome Sequencing

Whole genome sequencing (WGS) was conducted on whole blood‐derived DNA by contract sequencing centers from the NHLBI's Trans‐Omics for Precision Medicine (TOPMed) (Taliun et al. 2021). WGS data had an average of 39‐fold nuclear DNA coverage across TOPMed participants. The TOPMed Informatics Research Center harmonized all BAM files by aligning sequencing reads to the human genome build GRCh38 (Regier et al. 2018). In this study, the four study cohorts had WGS performed at three TOPMed sequencing centers, followed by joint variant identification using the Freeze 10 data (Table S1, Supporting Methods).

### 2.5 Identification of mtDNA Heteroplasmic Variants

The mitochondrial high‐performance call (*mitoHPC*) (Battle et al. 2022) was applied to mtDNA sequence from WGS data to identify mtDNA heteroplasmic variants and calculate mtDNA CN. mitoHPC is an automated pipeline to analyze mtDNA sequence reads with a circularized mitochondrial chromosome (chrM) (Battle et al. 2022). Briefly, mtDNA sequencing data was first mapped to chrM of GRCh38 (reference genome). Nuclear‐integrated mitochondrial sequence (NUMT) regions were extracted to build mtDNA read sinks. mtDNA reads were further remapped to a circularized chrM to recover low‐coverage areas.

Two iterations were performed to identify mtDNA heteroplasmic variants in mitoHPC: the first iteration identified mtDNA heteroplasmic variants with the revised Cambridge Reference Sequence (rCRS) as the reference genome; the second iteration generated a unique reference genome with “BCFtools” for each sample to lower false positives (Battle et al. 2022). We utilized the mtDNA heteroplasmic variants identified from the second iteration in subsequent analyses.

### 2.6 Calculation of mtDNA Aging Metrics

#### 2.6.1 Heteroplasmy Burden Scores of Rare Heteroplasmic Variants

In this study, a heteroplasmy variant was defined by a variant allele fraction (VAF) within 5%–95% in the same individual (Hong et al. 2023). Rare heteroplasmic variants were defined as those with a minor allele frequency (MAF) below 1% across all participants. We removed several specific sites that were previously identified as being associated with NUMTs or sequencing uncertainties due to their location within homopolymer regions (i.e., site positions: 1–61, 301, 302, 310, 316, 499, 567, 3107, 16088–16569). Additionally, sites with coverage below 250 were removed prior to the calculation of burden scores for rare variants, resulting in 16,015 mtDNA base positions used for the analysis. We used two rare variant‐based metrics to measure the total heteroplasmy level in each sample. MHcount, or heteroplasmy count, is the total number of rare heteroplasmic variants across *j* sites for *i* ^th^ individual: ${\text{MHcount}}_{i}=\sum_{j}H_{\mathrm{ij}}$. MSS, or mitochondrial local constraint score sum (Hong et al. 2023), is a weighted score based on the predicted functions of heteroplasmic variants: ${\mathrm{MSS}}_{i}=\sum_{j}m_{\mathrm{ja}}H_{\mathrm{ij}}$. $m_{\mathrm{ja}}$ measures mitochondrial local constraint (MLC) (Lake et al. 2024) for the alternative allele *a* at a heteroplasmy site *j*. MLC ranges from 0 to 1, with a higher MLC suggesting a more deleterious mutation. Therefore, higher MSS levels indicate more harmful biological consequences.

#### 2.6.2 Heteroplasmy Burden Score of Common Heteroplasmic Variants

In a recent study, 39 common heteroplasmic variants present in at least 500 individuals in UK Biobank were analyzed in genome‐wide association analysis (GWAS) (Gupta et al. 2023). We calculated the burden score of common heteroplasmic variants (MH_com_count) as the sum of 39 variants, ${\mathrm{MH}}_{\mathrm{com}}{\text{count}}_{i}=\sum_{j}C_{\mathrm{ij}}$, and tested the hypothesis that the burden of common heteroplasmic variants was not associated with epigenetic aging metrics.

#### 2.6.3 Estimation of mtDNA CN

The calculation of mtDNA CN was performed jointly by the TOPMed Informatics Research Center using WGS data. mtDNA CN was computed as a two times ratio of average mitochondrial genome coverage to average nuclear genome coverage by *mitoHPC*, considering the haploid nature of mtDNA. Average genome coverage was calculated as the number of mapped reads divided by the standard human genome size (Ding et al. 2015).

#### 2.6.4 Residuals of mtDNA Metrics

We obtained residuals for mtDNA metrics (i.e., MHcount, MH_com_count, MSS, and mtDNA CN) by regressing each metric on blood cell counts, batch effects, and smoking status in each cohort. We further standardized residuals of each mtDNA metric (mean = 0, standard deviation [SD] = 1) (Liu, Longchamps, et al. 2021).

### 2.7 Phenotype Description

To explore the relationship between mitochondrial aging metrics and EAAs on metabolic disorders, obesity and T2DM were used in the mediation analysis. Obesity was defined as a body mass index (BMI) ≥ 30 kg/m^2^. T2DM was defined as a fasting blood glucose level ≥ 126 mg/dL or current use of diabetes treatment (Liu, Longchamps, et al. 2021). We also assessed whether major CVD risk factors, BMI, hypertension, hyperlipidemia, T2DM, and prevalent CVD confounded the association between mtDNA metrics and EAAs. Stage 2 hypertension was defined as a systolic blood pressure ≥ 140 mmHg, diastolic blood pressure ≥ 90 mmHg, or current use of antihypertensive medications (Liu, Longchamps, et al. 2021). We focused on this group due to its clinical significance, higher risk of cardiovascular events, and well‐established diagnostic threshold. Hyperlipidemia was defined as a fasting total cholesterol ≥ 200 mg/dL, triglyceride ≥ 150 mg/dL, or current use of lipid‐lowering medications (Liu, Longchamps, et al. 2021). Prevalent CVD was defined as the presence of cardiovascular conditions, including recognized myocardial infarction, stroke, and congestive heart failure (Liu et al. 2023).

### 2.8 Association Analysis Between mtDNA and EAA Metrics

**FIGURE 1:**
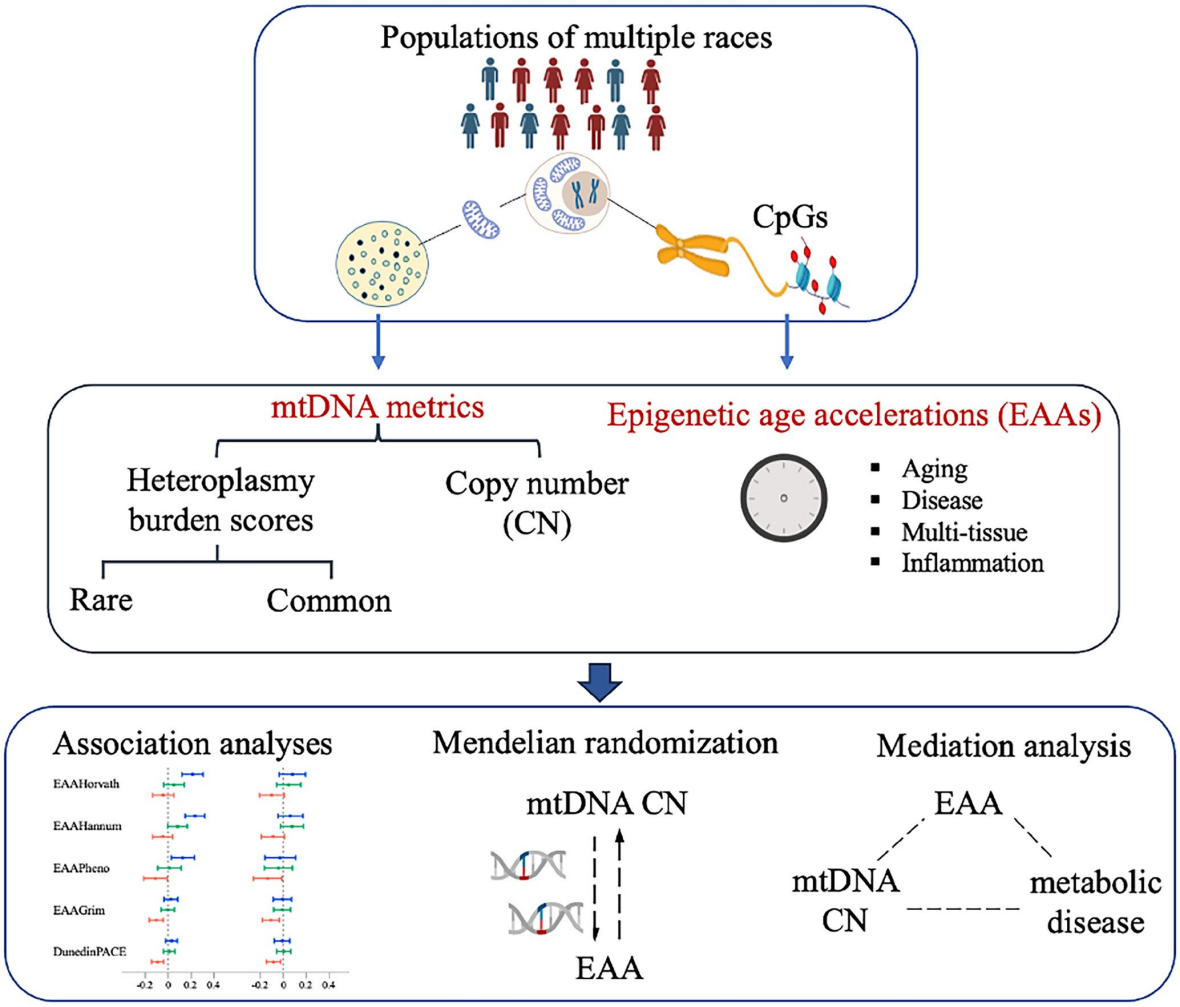
Study design flowchart. Mitochondrial DNA metrics include heteroplasmy burden scores and mtDNA copy number (mtDNA CN). Heteroplasmy burden scores were constructed based on rare and common heteroplasmic variants and functional prediction. Epigenetic age acceleration (EAA) metrics were calculated based on DNA methylation levels of specific CpGs. Association analyses of mtDNA metrics with EAAs were conducted, adjusting for covariates. We investigated if epigenetic aging mediated association between mtDNA CN and metabolic traits (e.g., obesity and type 2 diabetes mellitus) and assessed the causal relationship between mtDNA CN and EAAs.

Study participants with MHcount > 5 or mtDNA CN smaller than 40 were removed from downstream analyses to minimize potential sequencing error or sample contamination. In the primary analysis, cohort‐specific linear regression was performed to quantify the association of each EAA metric (dependent variables) with standardized residuals of each mtDNA metric (independent variables; i.e., MHcount, MSS, MH_com_count, and mtDNA CN), adjusting for chronological age, chronological age squared (Liu, Longchamps, et al. 2021), self‐identified sex, smoking status, proportion of white blood cell composition, batch effect, and lab index (in FHS) (Figure 1). Previous studies found that older participants had a lower level of mtDNA CN and a higher level of heteroplasmy burden compared to younger participants (Liu, Longchamps, et al. 2021; Liu, Fetterman, et al. 2021; Hong et al. 2023). Thus, stratified analyses were conducted by chronological age (i.e., < 60 and ≥ 60 years) and self‐identified sex groups in each cohort.

For all analyses, FHS was used as the discovery cohort, followed by replication analyses in CARDIA, JHS, and MESA. We assumed a single true underlying effect; therefore, a fixed‐effect inverse‐variance weighted (IVW) meta‐analysis was used to combine results across cohorts in both the overall and age‐specific samples.

#### 2.8.1 Sensitivity and Secondary Analyses

Previous studies demonstrated that mtDNA CN was associated with CVD (Liu et al. 2023) and cardiometabolic diseases (Liu, Longchamps, et al. 2021). To examine potential confounding effects of CVD and its risk factors on the associations between the EAA and mtDNA aging metrics, sensitivity analyses were performed by adjusting BMI, hypertension, hyperlipidemia, T2DM, and diagnoses of prevalent CVD as additional covariates. To evaluate whether maternal inheritance influenced the associations between the EAAs and mitochondria metrics, a linear mixed effects model with maternal structure as the random effect was performed in the FHS participants.

To study if the associations between mtDNA metrics and EAAs are different between race/ethnicity groups, we conducted a secondary meta‐analysis separately in self‐reported Black American and White American participants using fixed IVW meta‐analyses. Additionally, to identify which CpGs may contribute to the associations between EAAs and mtDNA metrics, we conducted secondary analyses between mtDNA metrics and the 1950 CpGs used to construct the EAA metrics in FHS participants.

### 2.9 Mediation Analysis of Aging Phenotypes

Previous meta‐analyses have reported associations of mtDNA CN with obesity and T2DM (Liu, Longchamps, et al. 2021), as well as associations between DNAm and these traits (Franzago et al. 2022; Miao et al. 2024; Li et al. 2025). Bi‐directional mediation analyses were conducted to investigate whether epigenetic aging (i.e., mediator) might mediate the association between mitochondrial health and metabolic traits in older participants (≥ 60 years), testing both directions by alternately modeling mtDNA CN and metabolic traits as predictor and outcome variables (Supporting Methods for details).

The natural indirect effect (NIE) (i.e., mediation effect), total effect (TE), and percentage mediated (PM) were estimated using two regression models:

Model 1: mediator ~ a×predictor+covariates

Model 2: $\text{outcome}\;\sim \;b\times \text{mediator}+c^{\prime }\times \text{predictor}+\text{covariates}$

Here, a represents regression coefficient for the predictor in Model 1; b and $c^{\prime }$ (i.e., the direct effect) represent the regression coefficients for the mediator and the predictor, respectively, in Model 2. NIE=a×b, $\mathrm{TE}=a\times b+c^{\prime }$, and $\mathrm{PE}=\left[\left(a\times b\right)/\left(a\times b+c^{\prime }\right)\right]\times 100\%$. Covariates included chronological age, chronological age squared, sex, smoking status, proportion of white blood cell composition, batch effect, and the lab index (in FHS). Mediation analysis was conducted using FHS as the discovery cohort, with JHS and MESA serving as replication cohorts.

### 2.10 Mendelian Randomization Analysis

Mendelian randomization (MR) uses single nucleotide polymorphisms (SNPs) identified in genome‐wide association studies (GWAS) to infer causal relationships in epidemiological research (Richmond and Davey Smith 2022). We applied bi‐directional two‐sample univariable Mendelian randomization MR (UVMR) to investigate the relationships between predictor (mtDNA CN) and outcome (EAAs) variables in the primary analyses. mtDNA CN‐associated SNPs (*p* < 5E‐8) were identified from a large GWAS (of 163,372 participants) (Gupta et al. 2023). SNPs were also identified in a GWAS of 34,710 participants for several non‐PC based DNAm aging metrics, including IEAA, EAAHannum, EAAPheno, and EAAGrim (McCartney et al. 2021). IEAA refers to an intrinsic epigenetic age acceleration, which measures the acceleration of Horvath age adjusting for cell counts of naive and exhausted CD8^+^ T cells, plasma B cells, CD4^+^ T cells, natural killer cells, monocytes, and granulocytes (Horvath et al. 2016). Independent SNPs were identified (linkage disequilibrium (LD) *r* ^*2*^ < 0.001 based on the 1000 Genomes phase 3 European reference panel) in each pair. The IVW method was used to combine the causal effect of individual SNPs in UVMR. Two sensitivity analyses (i.e., MR‐Egger and weighted‐median) were conducted to assess horizontal pleiotropy and to estimate the strengths of SNPs.

### 2.11 Software Package and Statistical Significance

All statistical analyses were performed using R (4.2.1). An association between mtDNA metrics and EAAs was considered significant at *p* < 0.01 (0.05/5). A false discovery rate (FDR) < 0.05 was applied in secondary analyses of individual CpGs. PC‐based clocks were calculated with “*run_calcPCClocks*” and “*run_calcPCClocks_Accel*” functions (https://github.com/MorganLevineLab/PC‐Clocks) (Higgins‐Chen et al. 2022), and DunedinPACE was calculated with the “*PACEProjector*” function in the “*DunedinPACE*” package (Belsky et al. 2022). Meta‐analyses were performed using the “*metagen*” function in the “*meta*” package (Guido et al. 2015). Mediation analyses were conducted using the “*regmedint*” function in the “*regmedint*” package (Yoshida et al. 2021). Two‐sample Mendelian randomization was performed with the “*twoSampleMR*” package (Hemani et al. 2017; Hemani et al. 2018).

## 3 Results

### 3.1 Sample Characteristics

**FIGURE 2:**
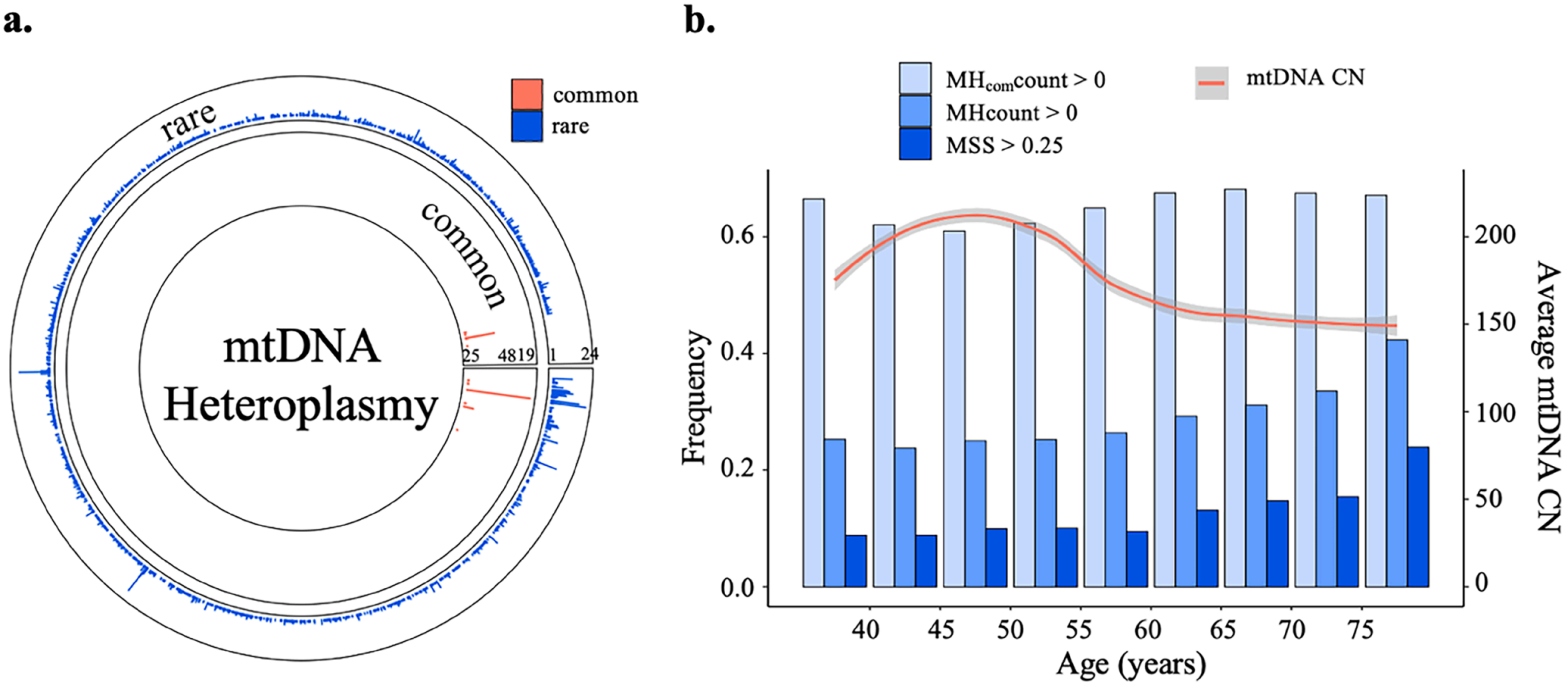
Distribution of heteroplasmy and mitochondrial DNA copy number (mtDNA CN). (a) Distribution of rare and common heteroplasmic variants across mtDNA base positions. Track 1 (outer) represents distribution and frequency (*y*‐axis: 0‐24) of rare heteroplasmic variants across positions (1‐16,569) in 6316 participants. Of those, 1489 mtDNA positions harbored at least one rare heteroplasmic variant (MAF < 0.01). Track 2 (inner) represents distribution and frequency (*y*‐axis: 25‐4819) of common heteroplasmic variants in 6316 participants. (b) Distribution of heteroplasmy burden and mtDNA CN across age groups. MH_com_count, burden score of common heteroplasmic variant count. MHcount, burden score of rare heteroplasmic variant count. MSS, burden score based on the predicted function of deleterious rare heteroplasmic variants.

This study included 6316 participants (58% women; 41% Black Americans) from CARDIA, FHS, JHS, and MESA (Table 1). Across participants, 1489 mtDNA positions harbored at least one rare heteroplasmic variant (MAF < 0.01), whereas 16 non‐overlapping mtDNA positions contained 39 common heteroplasmic variants used to calculate the common heteroplasmy burden score (Figure 2a).

Approximately 30% of participants carried at least one rare heteroplasmic variant, and roughly 10% of participants demonstrated a MSS score greater than 0.25. In contrast, about 60% of participants exhibited common heteroplasmic variants. For both MHcount and MSS, which were based on rare variants, we observed a crude positive correlation between the burden of these metrics and age, except for the younger CARDIA cohort. In contrast, such a trend was not observed for MH_com_count, which was based on common variants, in any cohort (Figure S1).

From age 55 onward, the proportion of participants with rare variant burden (MHcount > 0) increased, with a particularly pronounced rise observed for those carrying potentially deleterious variants (MSS > 0.25) (Figure 2b). The highest level of mtDNA CN was observed in participants aged 45–50 years, followed by a declining trend. Participants over 60 years had greater levels of MSS and MHcount, and lower mtDNA CN than younger participants (Figure 2b, Table S2). Thus, analyses were performed in the overall cohort samples and by age group (< 60 and ≥ 60 years) groups.

### 3.2 Associations Between mtDNA Heteroplasmy Burden Scores and EAAs

**FIGURE 3:**
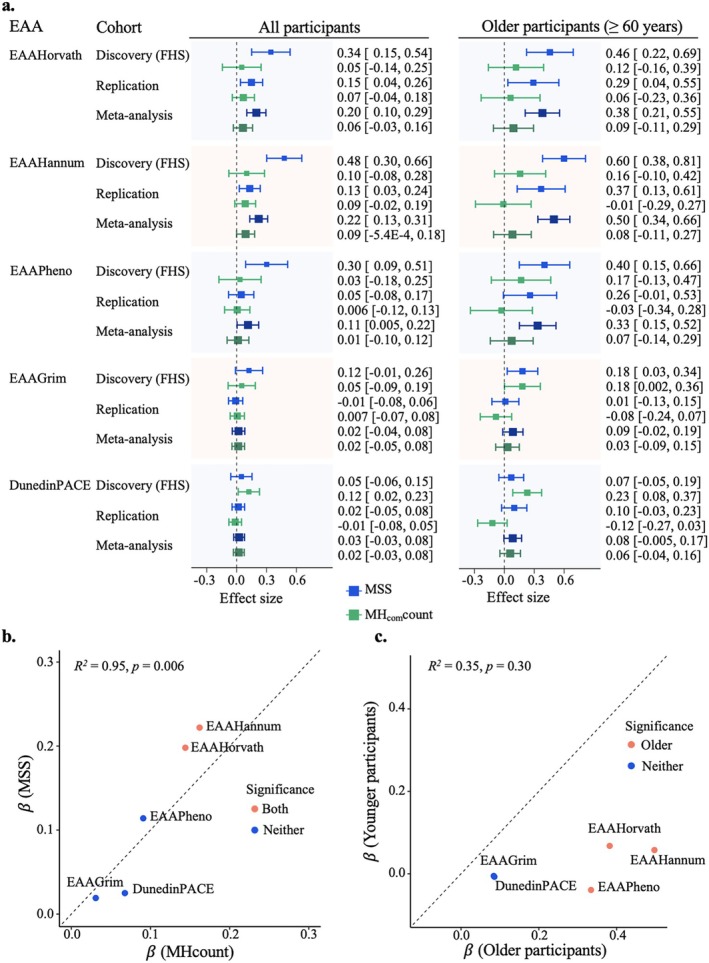
Association and meta‐analyses of mtDNA heteroplasmy burden with epigenetic age acceleration metrics. (a) Comparison of discovery, replication, and meta‐analyses of mitochondrial heteroplasmy burden score based on the predicted function of deleterious, rare heteroplasmic variants (MSS) and count score of common heteroplasmic variants (MH_com_count) with epigenetic age acceleration (EAA) metrics in the pooled sample (*n* = 6316) and older participants (≥ 60 years, *n* = 2000). (b) Correlation of effect sizes between MSS and count of rare heteroplasmic variants (MHcount) in the meta‐analysis of all samples, indicating consistent effect sizes between MSS and MHcount. (c) Correlation of effect sizes of MSS between younger (< 60 years) and older participants (≥ 60 years) in the meta‐analysis of all samples, indicating much larger effect sizes observed in older participants. In the meta‐analysis, we utilized the inverse variance weighted fixed‐effect meta‐analysis to summarize association analyses across four cohorts. The beta coefficients represented the change in EAA with one‐SD higher level of MSS and MH_com_count. The beta coefficients of DunedinPACE represented the change of 20‐year DunedinPACE with one‐SD higher level of MSS and MH_com_count.

We conducted association analysis between the heteroplasmy burden scores based on rare variants and PC‐based EAA metrics, adjusting for chronological age, chronological age squared (Liu, Longchamps, et al. 2021), self‐reported sex, smoking status, proportion of white blood cell composition, batch effect, and lab index (Figure 3a). The significance was considered with a threshold of *p* < 0.01. For the discovery analysis in FHS, MSS was positively associated with three EAAs (i.e., EAAHorvath, EAAHannum, EAAPheno). One‐SD higher level of MSS was associated with a 0.34‐year greater EAAHorvath (*p* = 4.8E‐4), a 0.48‐year greater EAAHannum (*p* = 1.2E‐7), and a 0.30‐year greater EAAPheno (*p* = 0.005). MSS also showed positive but non‐significant associations with EAAGrim (*β* = 0.12, *p* = 0.07) and DunedinPACE (*β* = 0.002, *p* = 0.38) (Table S3). The replication analyses were performed separately in CARDIA, JHS, and MESA, followed by a meta‐analysis across three cohorts. We observed consistent but slightly weaker associations between MSS and EAAHorvath (*β* = 0.15, *p* = 0.008), EAAHannum (*β* = 0.13, *p* = 0.01), and EAAPheno (*β* = 0.05, *p* = 0.48) in a meta‐analysis of the replication cohorts (Table S4). Similar to the discovery analysis, no significant association was observed between MSS and either EAAGrim or DunedinPACE in the meta‐analysis of the replication cohorts (Table S4). In the meta‐analysis of all samples from discovery and replication cohorts, one‐SD higher level of MSS was significantly associated with a 0.20‐year greater EAAHorvath (*p* = 5.5E‐5) and a 0.22‐year greater EAAHannum (*p* = 1.3E‐6), but was not associated with other EAAs (*p* = 0.04–0.55, greater than 0.01) (Table S5). The associations between MHcount and EAAs showed consistent directions and effect sizes (*R* ^2^ = 0.95) with those observed for MSS in the meta‐analysis of all samples (Figure 3b).

#### 3.2.1 Associations of Rare mtDNA Heteroplasmy Burden Score and EAAs by Age Groups

In the age‐group stratified analysis, stronger associations between MSS and EAAs were observed among the older participants (≥ 60 years) compared to younger participants (Figure 3a). Among older participants of FHS, greater effect sizes were observed between MSS and EAAHorvath (*β* = 0.46, *p* = 1.4E‐4), EAAHannum (*β* = 0.60, *p* = 6.1E‐8), EAAPheno (*β* = 0.40, *p* = 0.002), EAAGrim (*β* = 0.18, *p* = 0.02), and DunedinPACE (*β* = 0.003, *p* = 0.27) compared to younger participants (Figure 3c, Table S3). In the replication analyses, one‐SD increment of MSS was associated with a 0.37‐year greater EAAHannum (*p* = 0.003) among older participants, while such associations were not observed in younger participants of the three cohorts (Table S4). In the meta‐analysis across the four cohorts, one‐SD increment of MSS was associated with a 0.38‐year greater EAAHorvath (*p* = 1.4E‐5) in older participants, which was much stronger than that observed in younger participants (*β* = 0.07, *p* = 0.26) (Table S5). Similar to the findings in the full sample, a higher MSS burden score showed non‐significantly positive associations with EAAGrim (*β* = 0.09, *p* = 0.10) and DunedinPACE (*β* = 0.004, *p* = 0.06) in older participants. In contrast, among younger participants, no significant associations were observed between MSS and any of the EAA metrics (Tables S3–S5).

#### 3.2.2 Associations of Rare mtDNA Heteroplasmy Burden Score and EAAs by Sex

Because sex is a key component in GrimAge estimation (Lu et al. 2019), we performed sex‐stratified analysis to investigate whether associations between mtDNA heteroplasmy burden scores based on rare variants (MSS and MHcount) and PC‐based EAAGrim, as well as other EAAs, varied by sex. For EAAGrim, MSS showed a suggestive positive association in females from the discovery cohort (*β* = 0.26, *p* = 0.011), while weaker or null associations were observed in females in the meta‐analysis of replication cohorts. In contrast, EAAGrim showed predominantly non‐significant negative or null associations in males (Figure S2, Table S6). For other EAAs, including EAAHorvath, EAAHannum, EAAPheno, and DunedinPACE, higher MSS was associated with greater EAA levels in both females and males, although some of these associations did not reach statistical significance (Figure S2).

#### 3.2.3 Associations Between Common Heteroplasmic Variant Burden Score and EAAs

MH_com_count was calculated based on 39 common heteroplasmic variants (Gupta et al. 2023). No statistically significant associations with any EAAs were observed in the discovery, replication, or combined samples, nor in age‐ and sex‐stratified analyses (*p* ranges from 0.02 to 0.94) (Figure 3a, Figure S2, Tables S3–S6), except for a positive association between MH_com_count and DunedinPACE in older FHS participants (*β* = 0.01, *p* = 0.002). However, this association was not replicated in other cohorts or in meta‐analyses of the combined samples. Of the 39 heteroplasmic variants, 27 were insertions and deletions (INDELs) located in the D‐loop or homopolymer regions (i.e., POS: 300–317, 16180–16193 (Laricchia et al. 2022)), for which no MLC scores were available (Lake et al. 2024) (Table S7). Additionally, 12 of these 39 variants were assigned a low MLC score (Lake et al. 2024) (MLC < 0.1), indicating a low predicted deleteriousness.

#### 3.2.4 Sensitivity and Secondary Analyses

In sensitivity analyses, additionally adjusting for CVD and its risk factors strengthened associations between the heteroplasmy burden scores based on rare variants and the EAAs metrics (Tables S8–S10). For example, in FHS, a one‐SD increment of MSS was associated with higher EAAHorvath (*β* = 0.42, *p* = 1.6E‐5 vs. *β* = 0.34, *p* = 4.8E‐4), EAAHannum (*β* = 0.54, *p* = 4.0E‐10 versus *β* = 0.48, *p* = 1.2E‐7), and EAAPheno (*β* = 0.35, *p* = 6.3E‐4 vs. *β* = 0.30, *p* = 0.005) after additionally adjusting for CVD and its risk factors, compared to the models without this adjustment (Table S8). Including maternal structure as a random effect in the association testing yielded results highly consistent with the primary discovery analysis (*R* ^*2*^ = 0.995) (Figure S3). After adjusting for maternal structure, a positive association between MHcount and EAAGrim (*p* = 0.004) was observed (Table S11), whereas in the unadjusted analysis the association was in the same direction but did not reach statistical significance (*p* = 0.03) (Table S3).

In secondary analyses, we evaluated associations between heteroplasmy burden scores and EAAs between self‐identified Black and White American participants. Most associations displayed consistent direction between the two groups (Supplemental Results, Figure S4a, Table S5). Additional secondary analyses were conducted to further identify which CpGs contributed to the EAA associations with mtDNA burden scores in FHS. Of the 1950 distinct CpGs that were used to contract the EAA metrics, 85 were associated with MSS (Figure S5, Table S12), and 40 were associated with MHcount (FDR < 0.05) (Table S13), while no CpG probe was associated with MH_com_count.

## 4 Associations Between mtDNA CN and EAAs

**FIGURE 4:**
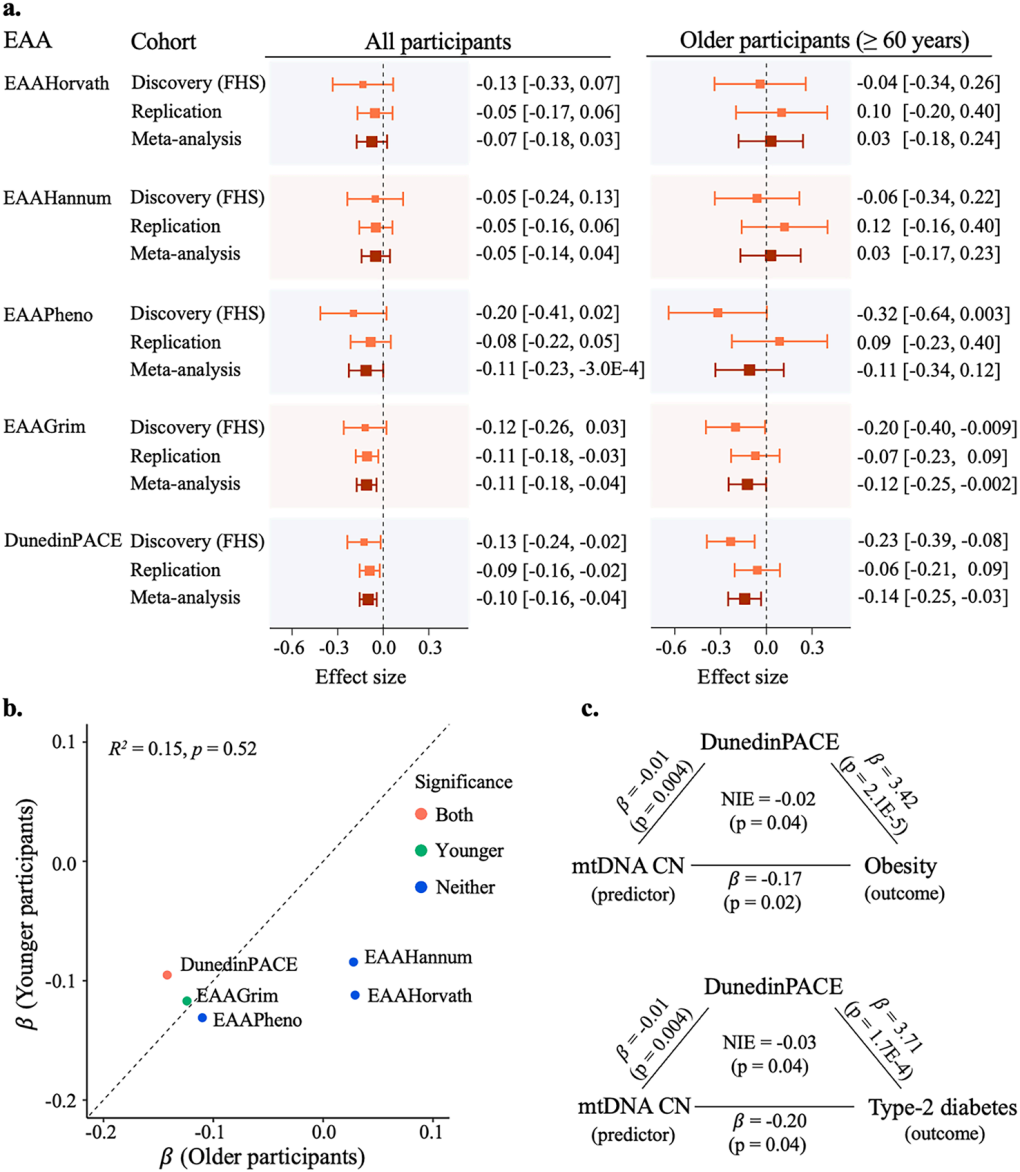
Association and meta‐analyses of mtDNA copy number with epigenetic age acceleration metrics. (a) Comparison of discovery, replication, and meta‐analyses of mtDNA copy number (mtDNA CN) with epigenetic age acceleration (EAA) metrics in the pooled sample (*n* = 6316) and older participants (≥ 60 years, *n* = 2000). (b) Correlation of effect sizes of mtDNA CN between younger (< 60 years) and older (≥ 60 years) participants in the meta‐analysis of all samples. (c) Mediation analysis of DunedinPACE on associations between mtDNA CN, obesity, and type 2 diabetes mellitus in older (≥ 60 years) FHS participants. In the meta‐analysis, we utilized the inverse variance weighted fixed‐effect meta‐analysis to summarize association analyses across four cohorts. The beta coefficients represented the change in EAA with one‐SD higher level of mtDNA CN. The beta coefficients of DunedinPACE represented the change of 20‐year DunedinPACE with one‐SD higher level of mtDNA CN.

We further investigated associations between mtDNA CN and EAAs (Figure 4a). We observed the inverse, although non‐significant (*p*: 0.02–0.58), associations between mtDNA CN and PC‐based EAAs in the discovery of FHS participants (Table S3). In the meta‐analysis of the three replication cohorts, inverse associations were also observed between the mtDNA CN and EAAs. For example, a one‐SD higher level of mtDNA CN was associated with a 0.11‐year lower EAAGrim (*p* = 0.005) and a 0.004‐year lower DunedinPACE (*p* = 0.008), but not significant for the other three EAAs (*p* > 0.2) (Table S4). In the meta‐analysis of the four cohorts, stronger associations were observed for EAAGrim (*β* = −0.11, *p* = 0.001) and DunedinPACE (*β* = −0.005, *p* = 6.0E‐4), and non‐significant for the other three EAAs (*p* ≥ 0.05) (Table S5). In stratified analyses by age groups, mtDNA CN showed significant associations with DunedinPACE in both younger (*β* = −0.005, *p* = 0.007) and older (*β* = −0.007, *p* = 0.01) participants, whereas a significant association between mtDNA CN and EAAGrim was observed only in the younger participants (*β* = −0.12, *p* = 0.004) (Figure 4b, Table S5).

In sex‐stratified analyses, higher mtDNA CN was generally associated with lower EAAs in females, particularly for EAAGrim and DunedinPACE, although not all associations reached significance. In males, associations were weaker and directionally inconsistent compared with those in females (Figure S6). For example, a one‐SD higher level of mtDNA CN was associated with a 0.12‐year lower EAAGrim (*p* = 0.003) and a 0.007‐year lower DunedinPACE (*p* = 2.2E‐4) in the meta‐analysis of all females, whereas such associations were non‐significant in males (EAAGrim: *β* = −0.09, *p* = 0.12; DunedinPACE: *β* = −0.004, *p* = 0.08) (Table S6).

In the sensitivity analysis, the adjustment of CVD and its risk factors greatly attenuated associations between the mtDNA CN and EAAs (Tables [Link], [Link]). mtDNA CN was not associated with any EAA in the discovery or replication analyses after adjusting for CVD adjustment. In the secondary analysis, the associations between mtDNA CN and EAAs displayed larger effect estimates in White American participants compared to Black American participants (Supplemental Results, Figure S4b, Table S5).

### 4.1 Mediation Analyses of the Relationship Between mtDNA CN and Metabolic Diseases, With EAAs as Mediators

We tested whether EAA metrics mediated the associations between mtDNA CN and two metabolic traits (i.e., obesity and T2DM), with predictor and outcome alternatively specified. In FHS older participants, mtDNA CN inversely associated with both obesity (*β* = −0.19, *p* = 9.7E‐3) and T2DM (*β* = −0.22, *p* = 0.02) (Table S14), consistent with previous results (Liu, Longchamps, et al. 2021). EAAPheno and DunedinPACE were positively associated with obesity (EAAPheno: *β* = 0.05, *p* = 0.02; DunedinPACE: *β* = 3.54, *p* = 9.6E‐6) and T2DM (EAAPheno: *β* = 0.05, *p* = 0.02; DunedinPACE: 3.86, *p* = 8.5E‐5). EAAGrim showed a suggestive positive association with obesity (*β* = 0.05, *p* = 0.08), but not with T2DM (*β* = 0.03, *p* = 0.43) (Table S14). Therefore, mediation analyses focused on EAApheno, EAAGrim, and DunedinPACE.

In FHS older participants, with mtDNA CN as the predictor, DunedinPACE mediated 11.1% (NIE = −0.02, *p* = 0.04) and 10.8% (NIE = −0.03, *p* = 0.04) of its associations with obesity and T2DM, respectively (Figure 4c), while EAAPheno and EAAGrim showed no significant mediation (percentage mediated < 5%, NIE = −0.003 to −0.1, and *p* > 0.1) (Table S15). Using obesity or T2DM as predictors and mtDNA CN as the outcome, DunedinPACE showed a similar proportion of mediation effect for both associations (NIE = −0.02 for both; 11.2% mediated for obesity, *p* = 0.06; and 12.5% mediated for T2DM, *p* = 0.05). No significant mediation effects of EAAPheno or EAAGrim were observed (*p* > 0.1) (Table S16). We conducted replication analyses in JHS and MESA older participants (≥ 60 years) (Tables S14–S16). In JHS, the indirect effects of DunedinPACE were near zero (*p* > 0.4) for both obesity and T2DM in both directions. In MESA, DunedinPACE showed indirect effects with direction and magnitude similar to those observed in FHS for both traits and both directions, although they were not statistically significant (*p* > 0.3) (Tables S15 and S16). In the meta‐analysis of mediation analysis across three cohorts, DunedinPACE showed a suggestive mediation effect in the associations of mtDNA CN (predictor) with obesity (NIE = −0.02, *p* = 0.06) and T2DM (NIE = −0.02, *p* = 0.08) (outcomes) (Figure S15). In the reverse direction, no suggestive mediation effects were observed from EAAPheno or EAAGrim (*p* > 0.1) (Figure S16).

### 4.2 Mendelian Randomization to Infer Causal Relationships Between mtDNA CN and EAAs

We conducted bi‐directional MR analyses to clarify the causal directionality among mtDNA CN and non‐PC based EAAs. To test the direction from mtDNA to EAAs, 41 independent SNPs (LD *r* ^*2*^ < 0.001) were selected from the GWAS of mtDNA CN (Table S17) (Gupta et al. 2023). MR‐median and MR‐Egger demonstrated weak evidence for a causal effect of mtDNA CN on EAAPheno (*β*_median_ = −0.66, *p* = 0.03) and EAAGrim (*β*_Egger_ = −0.88, *p* = 0.02), while IVW results were non‐significant (*p* > 0.05) (Table S18, Figure S7). Leave‐one‐out analyses indicated that no single SNP disproportionately influenced IVW (Figure S8) or MR‐Egger (Figure S9) methods. In MR‐median, two SNPs (rs11085147 and rs12247015) showed slight deviations in an ineffective direction, suggesting the robustness of MR estimates (Figure S10). To test the direction from non‐PC based EAAs to mtDNA CN, we identified various numbers of independent SNPs (LD *r* ^2^ < 0.001) for IEAA (*n* = 24), EAAHannum (*n* = 9), EAAPheno (*n* = 11), and EAAGrim (*n* = 4) from the GWAS of these EAAs (Tables S19–S22) (McCartney et al. 2021). No significant causal effects were observed from the EAA metrics to mtDNA CN using any of the MR methods (*p* ≥ 0.06) (Table S18).

## 5 Discussion

In this study, we examined the association between mitochondrial and epigenetic aging metrics in 6316 participants across four cohorts. We observed higher levels of mtDNA heteroplasmy burden (i.e., MHcount and MSS) and a lower level of mtDNA CN that were associated with accelerated epigenetic aging independent of age, and these associations showed a stronger magnitude among older participants. We also observed that DunedinPACE mediated a portion of the association between mtDNA CN and metabolite traits (obesity and T2DM) among older FHS participants. Our results reveal complex relationships among heteroplasmy, mtDNA CN, and EAAs, providing novel insights into the biological mechanisms underlying metabolic diseases.

We observed significant associations between heteroplasmy burden scores, MSS and MHcount, derived from rare heteroplasmic variants and several EAA metrics. In contrast, the burden score based on common heteroplasmic variants was not significant. The majority of these common heteroplasmic variants were INDELs located in the D‐loop and homopolymer regions, often lacking pathogenicity scores, while those with available scores had low predicted pathogenicity (MLC < 0.1). These findings indicate that common heteroplasmic variants are less likely to be associated with aging, consistent with a prior study showing that heteroplasmy in homopolymer regions has a much weaker association with age compared to those in non‐homopolymer regions (Battle et al. 2022). Together, these results support the interpretation that the common heteroplasmic variants are more likely to be of germline origin rather than somatic, while rare heteroplasmic variants appear to be more strongly associated with accelerated aging. In sex‐specific analyses, we observed a positive association between EAAGrim and MSS in females, whereas the association was negative in males. In contrast, the other EAAs showed consistent directions of association across both sexes. Different associations with EAAGrim between two sex groups were previously reported in lifestyle and cognition (O'Shea et al. 2022; Kim and Park 2025).

In this study, higher mtDNA CN was associated with lower levels of several EAA metrics. However, these associations were greatly attenuated after adjusting for CVD and its risk factors, suggesting these risk factors may confound the relationship between mtDNA CN and epigenetic aging. This interpretation aligns with previous studies showing that accelerated epigenetic aging is strongly correlated with CVD (Ashar et al. 2017; Liu et al. 2023; Sundquist et al. 2022) and major CVD risk factors, including obesity (Lundgren et al. 2022; Horvath et al. 2014; Li et al. 2024), T2DM (Vetter et al. 2023; Miao et al. 2024), hypertension (Kresovich et al. 2023), and hyperlipidemia (Gao et al. 2022). mtDNA CN has also been associated with white blood cell count, a common indication of CVD‐related inflammation (Vozarova et al. 2002; Dixon and O' Brien 2006; Ohshita et al. 2004; Orakzai et al. 2006; Karthikeyan and Lip 2006) as well as an indicator of chronic inflammation. Further, inflammation is highly related to biological aging (Baechle et al. 2023; Meier et al. 2023). Collectively, these findings suggest that mtDNA CN may primarily reflect an underlying inflammatory state associated with biological aging, rather than aging itself. In contrast, the associations between burden scores from rare heteroplasmic variants and EAA metrics were strengthened after adjusting for CVD‐related factors, suggesting that the heteroplasmy burden may serve as a more specific biomarker of biological aging. This interpretation aligns with the prevailing view that rare heteroplasmic variants arise from somatic mutations that accumulate over time (Stewart and Chinnery 2015; Wallace and Chalkia 2013), thereby more directly reflecting age‐related molecular changes.

While CVD risk factors may confound the observed cross‐sectional associations between mtDNA CN and EAA metrics, our bi‐directional mediation analysis suggests that DunedinPACE mediates the relationship between mtDNA CN and metabolic traits, such as obesity and T2DM in older FHS participants. However, our investigation could not determine the direction of the causal pathway because the indirect effects of DunedinPACE were in the same direction and of similar magnitude in both the forward direction (mtDNA CN as the predictor and metabolic traits as the outcomes) and the reverse direction (metabolic traits as the predictors and mtDNA CN as the outcome). These findings can be explained by the equivalent class of mediation analyses with reversing arrows (Thoemmes 2015), in which a nearly symmetric statistical decomposition likely exists in both directions (Maxwell et al. 2011). As a result, the indirect effects, calculated as the product of the exposure‐mediator effect and the mediator‐outcome effect, showed comparable magnitude and significance in both directions when using the cross‐sectional data.

In addition, because this study used cross‐sectional data, our findings highlight the need for future research in larger, ethnically diverse, longitudinal cohorts to robustly validate the mediation effects of DunedinPACE. Our two‐sample MR analysis provided potential evidence for a potential causal effect of mtDNA CN on epigenetic aging. Together, findings from confounding‐adjusted approaches, mediation analyses, and causal inference further highlight the complex relationships among mtDNA, aging, and metabolic health, and emphasize the importance of integrating multiple analytical methods in studying these relationships.

## 6 Limitation

In this study, we conducted multi‐level analyses across several cohorts to investigate the associations between mitochondrial and epigenetic aging metrics, leveraging genomic and metabolic data to explore their potential complex relationships. However, several limitations should be noted. First, we restricted our analysis to heteroplasmic variants with a MAF 5%–95% to minimize sequencing error and potential NUMT‐related confounding (Hong et al. 2023). This may have excluded true low‐level heteroplasmy and led to an underestimation of its associations with epigenetic aging. Additionally, the mitoHPC pipeline has limited sensitivity in detecting heteroplasmy in homopolymer (i.e., regions containing repeated sequences of identical nucleotides) or hypervariable regions (Battle et al. 2022). Our MR analysis employed GWAS SNPs associated with traditional (i.e., non‐PC based) EAA metrics, whereas our main analyses focused on PC‐based clocks. Although these two versions of clocks were highly correlated (*R* ^2^: 0.93–0.99 in FHS participants), the GWAS SNPs might not fully capture the genetic architecture of PC‐based clocks. The unavailability of a GWAS for DunedinPACE limited the exploration of causal pathways between mtDNA CN and DunedinPACE. Finally, the cohorts in this study differed in demographic characteristics, including age, sex, and race/ethnicity distributions. Although consistent procedures were applied to calculate EAAs and mtDNA metrics and to adjust for potential confounders, residual heterogeneity may still remain and introduce bias. Conversely, findings obtained from diverse populations may enhance the generalizability of our results to broader populations. In summary, we observed significant associations between mtDNA and epigenetic aging metrics, with evidence that EAA metrics mediate the association between mtDNA CN and metabolic diseases. These findings suggest the complex interplay between mtDNA metrics, epigenetic pathways, and metabolic traits, advancing our understanding of aging mechanisms by linking nuclear and mitochondrial genomes to clinical outcomes. However, large, diverse, longitudinal studies are needed to clarify the directionality of these relationships.

## Author Contributions

Chunyu Liu and Mengyao Wang conceived and designed the study. Mengyao Wang and Yinan Zheng performed data analysis. Mengyao Wang, Yinan Zheng, Meng Lai, Emmanuel Saake, Xue Liu, and Xiuqing Guo contributed to data preprocessing and quality control. Nicole J. Lake contributed to heteroplasmy functional scoring. Stephen S. Rich, Jerome I. Rotter, Yongmei Liu, Laura M. Raffield, April P. Carson, Myriam Fornage, Lifang Hou, and Daniel Levy provided funding for sequencing and/or DNA methylation data. Mengyao Wang and Chunyu Liu wrote the manuscript. Dan E. Arking, Christina A. Castellani, Jiantao Ma, Drew R. Nannini, Kai Zhang, Kent D. Taylor, Tianxiao Huan, and Roby Joehanes provided critical review of the manuscript. Chunyu Liu supervised the project and secured funding for statistical analyses. All authors reviewed and approved the final manuscript.

## Conflicts of Interest

Laura M. Raffield is a consultant for the TOPMed Administrative Coordinating Center (through Westat). The remaining authors declare no competing interests.

## Supporting information


**Data S1:** acel70279‐sup‐0001‐DataS1.xlsx.

**Appendix S1:** acel70279‐sup‐0002‐AppendixS1.pdf.

## Data Availability

The whole genome sequencing (WGS), DNA methylation, and phenotypic data from the Coronary Artery Risk Development in Young Adults Study (CARDIA), Framingham Heart Study (FHS), the Jackson Heart Study (JHS), and Multi‐Ethnic Study of Atherosclerosis Study (MESA) Cohort, used for association analysis of mitochondrial DNA metrics and DNA methylation acceleration metrics, are available in the dbGaP database under accession codes phs001612.v3.p3 (CARDIA) [https://www.ncbi.nlm.nih.gov/projects/gap/cgi‐bin/study.cgi?study_id=phs001612.v3.p3], phs000007.v32.p13 (FHS) [https://www.ncbi.nlm.nih.gov/projects/gap/cgi‐bin/study.cgi?study_id=phs000007.v34.p15], phs000964 (JHS) [https://www.ncbi.nlm.nih.gov/projects/gap/cgi‐bin/study.cgi?study_id=phs000964.v5.p1], and phs001416.v1.p1 (MESA) [https://www.ncbi.nlm.nih.gov/projects/gap/cgi‐bin/study.cgi?study_id=phs001416.v1.p1]. The WGS, RNA‐seq, and phenotypic data from all cohorts used in this study are available under restricted access to ensure confidentiality and protect participant privacy. All summary data generated in this study are available in the main manuscript and Supporting Information within this paper.
