# Supplementary material for: Association of Epigenetic Age Acceleration and Mitochondrial DNA‐Based Aging Metrics Provides Insights Into Mechanisms of Aging‐Related Diseases
Source: Aging Cell. 2025 Oct 24;24(12):e70279. doi: 10.1111/acel.70279 (PMC12686565; doi:10.1111/acel.70279)
Supplement: Supplementary file 2 — Appendix S1: acel70279‐sup‐0002‐AppendixS1.pdf. [file ACEL-24-e70279-s001.pdf]

SUPPORTING INFORMATION

Association of Epigenetic Age Acceleration and Mitochondrial DNA-based Aging Metrics Provides Insights into Mechanisms of Aging-Related Diseases

Mengyao Wang, Yinan Zheng, Meng Lai, Emmanuel Saake, Xue Liu, Xiuqing Guo, Kent D. Taylor, Tianxiao Huan, Roby Joehanes, Drew R. Nannini, Kai Zhang, Nicole J. Lake, Christina A. Castellani, Stephen S. Rich, Jerome I. Rotter, Yongmei Liu, Laura M. Raffield, April P. Carson, Myriam Fornage, Jiantao Ma, Dan E. Arking, Lifang Hou, Daniel Levy, Chunyu Liu

Email: liuc@bu.edu

CONTENTS

Cohort acknowledgements	Page 3
Supplementary methods	Page 5
Supplementary results	Page 11
Supplementary figures	Page 12
References	Page 23

COHORT ACKNOWLEDGEMENTS

The CARDIA is conducted and supported by the National Heart, Lung, and Blood Institute (NHLBI) in collaboration with the University of Alabama at Birmingham (75N92023D00002 & 75N92023D00005), Northwestern University (75N92023D00004), University of Minnesota (75N92023D00006), and Kaiser Foundation Research Institute (75N92023D00003). CARDIA was also supported in part by the Intramural Research Program of the National Institute on Aging (NIA) and an intra-agency agreement between NIA and NHLBI (AG0005). The DNA methylation laboratory work and analytic component were funded by the American Heart Association (17SFRN33700278 and 14SFRN20790000, Northwestern University, to Dr Hou) and NIA R21AG068955 (to Drs Liu and Zheng). The authors would like to thank all staffs and participants involved in the CARDIA study.

The FHS was supported by NIH contracts N01-HC-25195, HHSN268201500001I, and 75N92019D00031. Genome sequencing for “NHLBI TOPMed: Whole Genome Sequencing and Related Phenotypes in the Framingham Heart Study” (phs000974.v1.p1) was performed at the Broad Institute Genomics Platform (3R01HL092577-06S1, 3U54HG003067-12S2). The DNA methylation data was provided by NHLBI’s Systems Approach to Biomarker Research in Cardiovascular Disease Initiative (SABRe CVD Initiative) and sponsored by the NIH intramural fund. This research is also supported by the National Heart, Lung and Blood Institute and grant supplement R01-HL092577-06S1. Authors thank the FHS study participants for their dedication.

The JHS is supported and conducted in collaboration with University of Mississippi Medical Center (HHSN268201800010I, HHSN268201800011I and HHSN268201800012I), Jackson State University (HHSN268201800013I), Tougaloo College (HHSN268201800014I), and the Mississippi State Department of Health (HHSN268201800015I) contracts from the National Heart, Lung, and Blood Institute (NHLBI) with additional support from the National Institute on Minority Health and Health Disparities (NIMHD). Genome sequencing for “NHLBI TOPMed: The Jackson Heart Study” (phs000964.v1.p1) was performed at the Northwest Genomics Center (HHSN268201100037C). The phenotype harmonization, data management, sample-identity QC, and general program coordination were provided by the TOPMed Data Coordinating Center (R01HL-120393; U01HL-120393; contract HHSN268201800001I). The views expressed in this manuscript are those of the authors and do not necessarily represent the views of the National Heart, Lung, and Blood Institute; the National Institutes of Health; or the U.S. Department of Health and Human Services.

In MESA, whole genome sequencing (WGS) was supported by the TOPMed program by NHLBI. WGS for “NHLBI TOPMed: Multi-Ethnic Study of Atherosclerosis (MESA)” (phs001416.v1.p1) was performed at the Broad Institute of MIT and Harvard (3U54HG003067-13S1). Centralized read mapping and genotype calling, along with variant quality metrics and filtering were provided by the TOPMed Informatics Research Center (3R01HL-117626-02S1). Phenotype harmonization, data management, sample-

identity QC, and general study coordination, were provided by the TOPMed Data Coordinating Center (3R01HL-120393-02S1). MESA and the MESA SHARe project are conducted and supported by the National Heart, Lung, and Blood Institute (NHLBI) in collaboration with MESA investigators. Support for MESA is provided by contracts 75N92025D00022, 75N92020D00001, HHSN268201500003I, N01-HC-95159, 75N92025D00026, 75N92020D00005, N01-HC-95160, 75N92020D00002, N01-HC-95161, 75N92025D00024, 75N92020D00003, N01-HC-95162, 75N92025D00027, 75N92020D00006, N01-HC-95163, 75N92025D00025, 75N92020D00004, N01-HC-95164, 75N92025D00028, 75N92020D00007, N01-HC-95165, N01-HC-95166, N01-HC-95167, N01-HC-95168, N01-HC-95169, UL1-TR-000040, UL1-TR-001079, UL1-TR-001420, UL1TR001881, and R01HL105756. The authors thank the MESA participants and the MESA investigators and staff for their valuable contributions. A full list of participating MESA investigators and institutes can be found at http://www.mesa-nhlbi.org.

SUPPLEMENTAL METHODS

Coronary Artery Risk Development in Young Adults Study (CARDIA) (n = 2,289)

Study description

The CARDIA is a bi-racial (Black or White Americans) and multi-center longitudinal study initiated in 1985 to investigate risk factors for developing cardiovascular diseases in early adulthood(Lloyd-Jones et al. 2021). The CARDIA enrolled 5,115 young participants (51.6% Black Americans, 18-30 years old) at the baseline in four areas: Birmingham, Chicago, Minneapolis, and Oakland. With 35 years following, CARDIA has already conducted 9 follow-up examinations. This study included 2,289 CARDIA participants with both mitochondrial DNA (mtDNA) and DNA methylation (DNAm) measurements.

Whole genome sequencing

The DNA in CARDIA (phs001612) was sequenced at the Baylor Human Genome Sequencing Center (HHSN268201600033I). Centralized read mapping and genotype calling, along with variant quality metrics and filtering were provided by the TOPMed Informatics Research Center (3R01HL-117626-02S1). Phenotype harmonization, data management, sample-identity QC, and general study coordination, were provided by the TOPMed Data Coordinating Center (3R01HL-120393-02S1). Whole genome sequencing was used for estimating mitochondrial DNA copy number (mtDNA CN) and mtDNA heteroplasmy.

DNA methylation measurement

DNA methylation was analyzed using the Infinium MethylationEPIC BeadChip (EPIC array) in whole blood. Quality control and preprocessing were performed with the R package ENmix using default settings. Low-quality methylation measurements were identified and excluded based on low detection p-values (< 1.0E-6) or few bead counts (< 3). We removed 6,209 CpG sites with a low detection rate (< 95%) and 87 samples with over 5% low-quality measurements or very low bisulfite conversion probe intensity. After this, 95 extreme outlier samples, defined by Tukey's method, were also excluded. The remaining samples underwent ENmix preprocessing, including background correction and dye bias correction using RELIC. M and U intensities were separately quantile-normalized for Infinium I and II probes. Low-quality methylation values and extreme β-value outliers were set as missing.

Statistical analysis

The epigenetic age acceleration (EAA) of PC-based clocks was attained by regressing each PC-based clock on age in each race/ethnicity group (i.e., White American and Black American). Standardized residuals of mtDNA metrics (i.e., MSS, MHcount, MH_{com}count, and mtDNA CN) were attained by first regressing each mtDNA metric on blood draw year, smoking status, and estimated blood cell proportions, then applying the standardization (mean=0, SD=1). The association analysis between each EAA and standardized residuals of each mtDNA metric was conducted in White Americans and Black Americans separately, adjusting for age, age-squared, sex,

smoking status, proportion of estimate white blood cells (i.e., CD8T, CD4T, natural killer, monocyte, granulocyte, and B cell) and batch effects (i.e., chip, row, and column). The fixed-effect inverse variance weighted (IVW) meta-analysis was applies to estimate association results for the pooled sample.

The Framingham Heart Study (FHS) (n = 1,745)

Study description

The FHS is a longitudinal study to investigate risk factors for cardiovascular diseases. The FHS has been recruited and followed three generations (n total ~ 15,000) of participants since 1948(Dawber et al. 1951). This study utilized 1,292 Offspring cohort participants at the 8th exam (2005-2008) and 453 Third Generation cohort participants at the 2nd exam (2008-2011) with both mtDNA and DNAm measurements(Feinleib et al. 1975; Splansky et al. 2007). Health exams were conducted regularly (every four to eight years) to collect demographic characteristics and cardiovascular risk factors (e.g., blood pressure, alcohol consumption, etc.)(Andersson et al. 2019). All the FHS participants in this study were self-reported White Americans.

Whole genome sequencing

The DNA in FHS (phs000974) was sequenced at Broad Institute of MIT and Harvard (3R01HL092577-06S1 and 3U54HG003067-12S2). This research is supported by the National Heart, Lung and Blood Institute and grant supplement R01-HL092577-06S1 through contracts NO1-HC-25195, HHSN268201500001I and 75N92019D00031. Whole-genome sequencing data were jointly processed for read mapping and genotype calling, with variant quality metrics and filtering provided by the TOPMed Informatics Research Center (3R01HL-117626-02S1). mtDNA CN and mtDNA heteroplasmy were estimated and identified using whole genome sequencing.

DNA methylation measurement

DNA methylation profiling and quality control procedures in FHS were previously described(Liu et al. 2018). DNA methylation of 2,846 Offspring participants attending the 8th exam and 1549 Third Generation participants attending the 2nd exam was sponsored by the NHLBI intramural funds awarded to Daniel Levy. Gentra Puregene DNA extraction kit (Qiagen, Venlo, Netherlands) was used for genomic DNA extraction from buffy coat. EZ DNA Methylation Kit (Zymo Research, Irvine, CA) was used for bisulfite conversion of DNA. DNA methylation levels were measured using Infinium HumanMethylation450 BeadChip array (Illumina Inc, San Diego, CA). The DNA methylation measurement was processed in three labs. Methylation levels of 576 Offspring samples, which were included in a previous case-control study of cardiovascular disease (ANNOtate), was measured at one lab. The remaining 2,270 Offspring samples from the Offspring cohort were analyzed at another lab. Methylation levels of 1,549 Third Generation samples was measured at the Illumina lab. Total probe intensity and the methylated probe intensity were obtained using Illumina Genome Studio (version 2011.1) and methylation module (version 1.9.0). The methylation beta

was defined as follows: $= \frac{M}{\mathrm{M+U+100}}$. Here M was the methylated signal and U was the unmethylated signal. To restrict technical artifacts, we used the DASEN method in wateRmelon in software R to normalize the resulting betas. Several quality control procedures were applied to DNA methylation data. At CpG level, cross-reactive probes mapping to multiple locations were removed(Chen et al. 2013). Low quality probes were removed, including probes with high missing rate (> 20%), with SNPs at CpG sites (MAF>5% in EUR 1000G), or with ≤ 10 base pair single base extension(Chen et al. 2013; Kuan et al. 2010). At participant level, we excluded low-quality samples including samples with high missing rate (> 1%), with outliers identified by multi-dimensional scaling (MDS) analysis(Taguchi & Oono 2005), or poor match to the 65 SNP genotypes between the Infinium HumanMethylation 450K BeadChip array and previous genotyping or 1000 Genome imputation.

Statistical analysis

Lab-specific EAA of each PC-based clock was calculated by regressing each PC-based clock on age. Standardized residuals of mtDNA metrics (i.e., MSS, MHcount, MH_{com}count, and mtDNA CN) were calculated by regressing each mtDNA metric on blood draw year, smoking status, and estimated cell counts (i.e., white blood cell, platelet, and neutrophil) in the pooled sample. The association analysis was conducted between epigenetic aging metrics and standardized residuals of each mtDNA metric, adjusting for age, age-squared, sex, smoking status, estimated blood cell proportions (i.e., CD4T, CD8T, monocyte, granulocyte, natural killer, and B cell), batch effects (i.e., chip, row, column), and the lab index.

The Jackson Heart Study (JHS) (n = 1,424)

Study description

The JHS(Sempos et al. 1999) is one of the largest community-based longitudinal African American cohort studies initiated in 1998 to investigate the cause of cardiovascular disease. The JHS originally recruited 5,306 African Americans residing in Jackson, Mississippi, metropolitan area (2000-2004). Till 2015, JHS has implemented two follow-up exams (2005-2008 and 2009-2012) so far. This study utilized 1,424 JHS participants from the baseline exam with DNAm data collected at the same time(Wilson et al. 2005).

Whole genome sequencing

Whole genome sequencing for JHS (phs000964) was performed in the Nickerson Laboratory at University of Washington (HHSN268201100037C). Whole genome sequence data are available for 3,406 participants after quality control. Core support including centralized genomic read mapping and genotype calling, along with variant quality metrics and filtering were provided by the TOPMed Informatics Research Center (3R01HL-117626-02S1; contract HHSN268201800002I). Whole genome sequencing data were used to identify mtDNA heteroplasmy and estimate mtDNA CN.

DNA methylation measurement

The Jackson Heart Study methylation level was measured by the Illumina EPIC (850k) array. The data was normalized using the minfi R package, and samples that did not pass quality control were removed by Steve Horvath's group prior to adjustment for technical variation/batch effects. Noob normalization and generation of methylation beta values from both ASN0104 and ASN0148 was performed by Steve Horvath's lab. Sample outliers were identified based on hierarchical clustering and removed. The dataset used in this study "jhs_betas_combat_adjusted" was adjusted for known batch effects (group, plate, well) using the SVA R package v3.30.1 ComBAT function. This method uses surrogate variable analysis to perform a global data adjustment based on batch effects. In this dataset, there are 866836 probes and 1706 individuals. JHS participants have approximately 83% mean similarity to 1000G AFR reference panels based on prior work, but no participants were excluded based on ancestry proportions.

Statistical analysis

The EAA of each PC-based clock was calculated by regressing each PC-based clock on age. Standardized residuals of mtDNA metrics (i.e., MSS, MHcount, MH_{com}count, and mtDNA CN) were calculated by regressing each mtDNA metric on blood draw year, smoking status, and estimated cell counts (i.e., white blood cell, platelet, and lymphocyte) in the pooled sample. The association analysis was conducted between epigenetic aging metrics and standardized residuals of each mtDNA metric, adjusting for age, age-squared, sex, smoking status, estimated blood cell proportions (i.e., CD4T, CD8T, monocyte, granulocyte, natural killer, and B cell), and batch effects (i.e., chip, row, column).

Multi-Ethnic Study of Atherosclerosis Study (MESA) (n = 858)

Study description

The MESA(Bild 2002; Blaha & DeFilippis 2021) is a multi-ethic and multi-center longitudinal study initiated in 2000 to investigate preclinical cardiovascular diseases. At baseline, MESA recruited 6,814 participants (38% European, 28% African, 22% Hispanic, and 12% East Asian Americans) from 6 centers: Columbia University, Johns Hopkins University, Northwestern University, University of California Los Angeles, University of Minnesota, Wake Forest University(Blaha & DeFilippis 2021). To date, 6 follow-up exams have already been implemented to track changes in risk factors and incorporate new measurements where ethnic differences could be detected under a multi-ethnic nature. This study utilized 858 MESA participants available at baseline exam (2000-2002) with accessible mtDNA and DNAm measurements.

Whole genome sequencing

WGS for the TOPMed program was supported by the NHLBI. WGS for the NHLBI's TOPMed (phs001416.v1.p1) was performed at the Broad Institute of MIT and Harvard (3U54HG003067-13S1) using the Affymetrix Genome-Wide Human SNP Array 6.0.. Centralized read mapping and genotype calling, along with variant quality metrics and filtering were provided by the TOPMed Informatics Research Center (3R01HL-117626-02S1). Phenotype harmonization, data management, sample-identity QC, and

general study coordination, were provided by the TOPMed Data Coordinating Center (3R01HL-120393-02S1).

DNA methylation measurement

DNA methylation of MESA was sponsored by NHLBI's TOPMed program (phs001416.v1.p1), contract HHSN268201600034I. Methylation level was assessed using the Illumina Infinium HumanMethylationEPIC BeadChip. The methylation beta was estimated as follows: $= \frac{M}{\mathrm{M+U+100}}$. Here M was the methylated signal and U was the unmethylated signal, Watermelon package and minfi(v1.22.1) package were used to process the raw data (.idat). To perform background correction and normalization, we used normal-exponential convolution with out-of-band Infinium I probes (noob). The sample concordance was checked by comparing the genotypes identified in previous genotyping arrays to the 59 SNPs in the methylation data (Details of the profiling and quality control procedure can be found in https://www.nhlbiwgs.org/sites/default/files/TOPMed_Methylation_array_pipeline_CORE yr3.pdf). Sex mismatch samples were removed, and probes with detection p-values > 0.05 were assigned value of NA.

Statistical analysis

Ancestry-specific EAA of each PC-based clock was calculated by regressing each PC-based clock on age. Standardized residuals of mtDNA metrics (i.e., MSS, MHcount, MH_{com}count, and mtDNA CN) were calculated by regressing each mtDNA metric on blood draw year, smoking status, and estimated blood cell proportions in each ancestry group. The association analysis was conducted between epigenetic aging metrics and standardized residuals of each mtDNA metric, adjusting for age, age-squared, sex, the ancestry index, smoking status, estimated blood cell proportions (i.e., CD4T, CD8T, monocyte, neutrophil, eosinophil, natural killer, and B cell), and batch effects (i.e., chip, row, column). The association results of the pooled sample were estimated with a fixed-effect IVW meta-analysis across ancestry groups.

Mediation analysis

We conducted bi-directional mediation analyses to assess whether EAAs mediated the associations between mtDNA copy number (mtDNA CN) and metabolic traits in older participants (≥60 years), testing both directions by alternately treating mtDNA CN and metabolic traits as the predictor and outcome (diagram below).

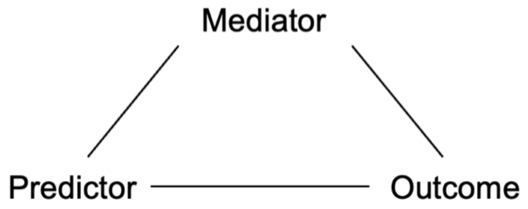

The indirect (mediation) effect was estimated using two regression models:
Model 1: $mediator \sim a \times predictor + covariates$

Model 2: $outcome \sim b \times mediator + c' \times predictor + covariates$

Here, a represents the regression coefficient for the predictor in Model 1; b and c' represent regression coefficients for the mediation and the predictor, respectively, in Model 2. We used natural indirect effect (NIE) to quantify the mediation of mediator on the association between the predictor and the outcome. The $NIE = a \times b$, $total\ effect = a \times b + c'$, and $percentage\ mediated = (a \times b)/(a \times b + c')$. Covariates included chronological age, chronological age squared, sex, smoking status, proportion of white blood cell composition, batch effect, and the lab index (in FHS). Mediation analysis was conducted using FHS as the discovery cohort, with JHS and MESA serving as replication cohorts.

For the forward direction, we tested whether EAAs mediated an association between mtDNA CN as the predictor and metabolic traits (i.e., obesity and type-2 diabetes mellitus (T2DM)) as the outcome.

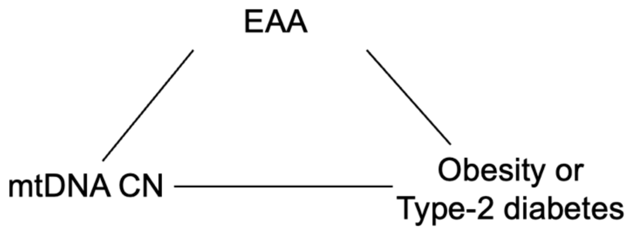

Model 1: $EAA \sim a \times mtDNA\ CN + covariates$
Model 2: $Obesity/T2DM \sim b \times EAA + c' \times mtDNA\ CN + covariates$
$NIE(mediation) = a \times b$
$total\ effect = a \times b + c'$
$percentage\ mediated = (a \times b)/(a \times b + c')$

In the backward direction, we tested whether EAAs mediated an association between metabolic traits as the predictor and mtDNA CN as the outcome.

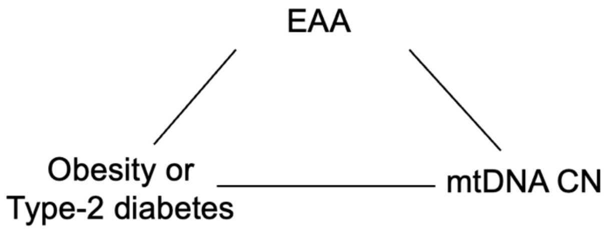

Model 1: $EAA \sim d \times obesity/T2DM + covariates$
Model 2: $mtDNA\ CN \sim e \times EAA + f' \times obesity/T2DM + covariates$
$NIE(mediation) = d \times e$
$total\ effect = d \times e + f'$
$percentage\ mediated = (d \times e)/(d \times e + f')$

SUPPLEMENTAL RESULTS

Associations of mtDNA heteroplasmy with epigenetic aging in race/ethnicity groups

A higher proportion of White Americans was observed in both age groups than the proportion of Black Americans (younger: WA vs. AA 53.4% vs. 46.6%; older: WA vs. AA 68.9% vs. 31.2%). The associations in the meta-analysis across 4 cohorts display consistent and positive directionality in both White and Black Americans, despite the difference in the magnitude of associations (R^2 of beta estimates=0.47, p=0.20) (**Figure S2a**). One-SD higher level of MSS was associated with a 0.29-year greater EAAHorvath (p=5.1E-6) and a 0.33-year greater EAAHannum (p=2.9E-8) in White Americans, while no significant association was observed in Black Americans (p in 0.41 – 0.93) (**Table S5**). MHcount showed similar results as MSS, that is, we observed stronger associations of MHcount with epigenetic aging metrics, including EAAHorvath and EAAHannum, in White Americans than in Black Americans (**Table S5**).

No association of MH_{com}count was observed with any epigenetic aging metric in Black Americans or White Americans (**Table S5**).

Associations of mtDNA CN with epigenetic aging in race/ethnicity groups

In ancestry-specific analyses, stronger associations were observed in White Americans than in pooled samples, while no significant associations in Black Americans (R^2 of beta estimates=0.07, p=0.66) (**Figure S2b**). In the meta-analysis across four cohorts, DunedinPACE decreased a 0.008-year (p=7.4E-6) in White Americans with one-SD increment of mtDNA CN, compared to a 0.005-year (p=6.0E-4) decline in pooled samples (**Table S5**).

SUPPLEMENTAL FIGURES

Figure S1. Distributions of mitochondrial heteroplasmic burden score across age in each cohort

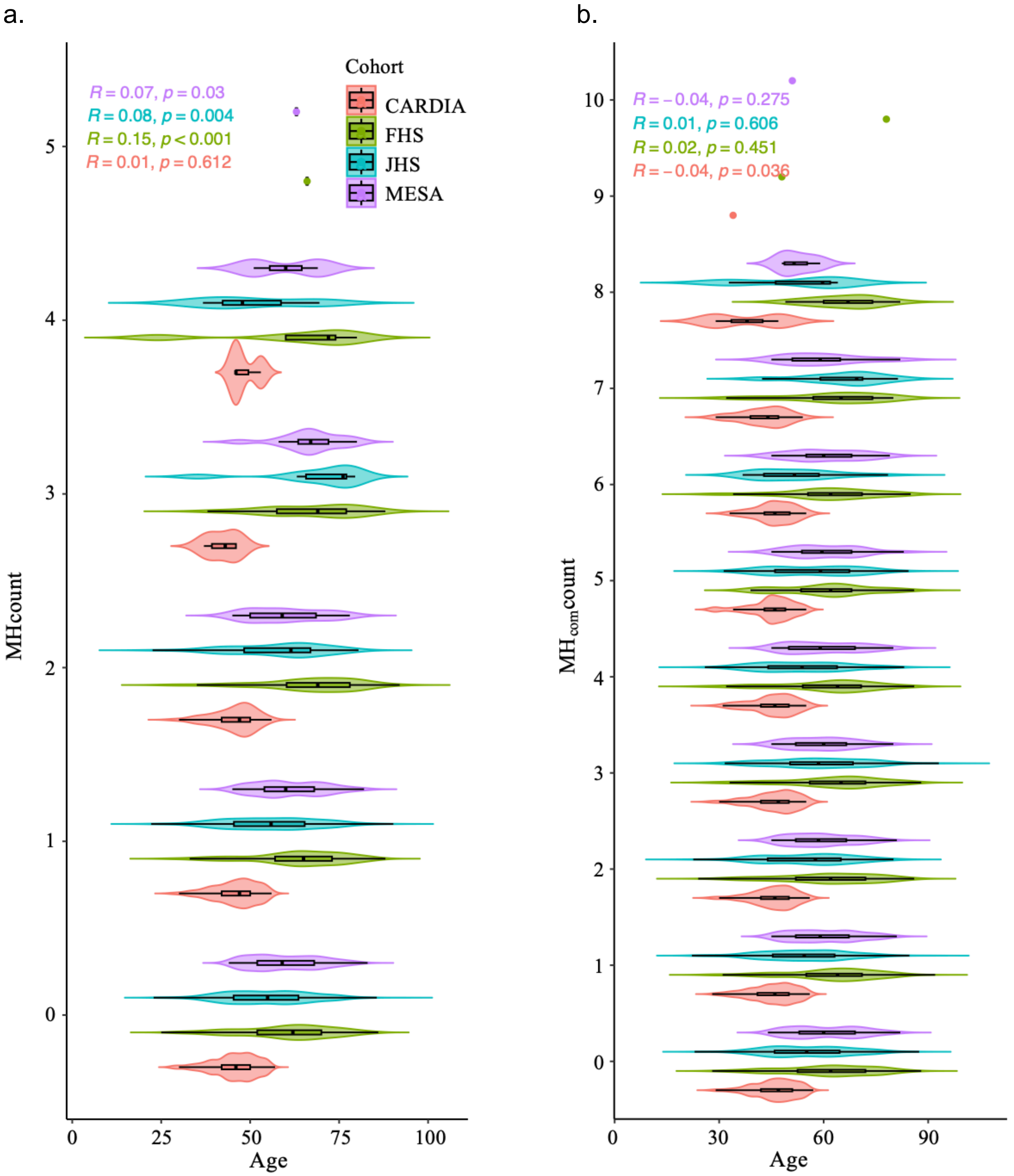

c.

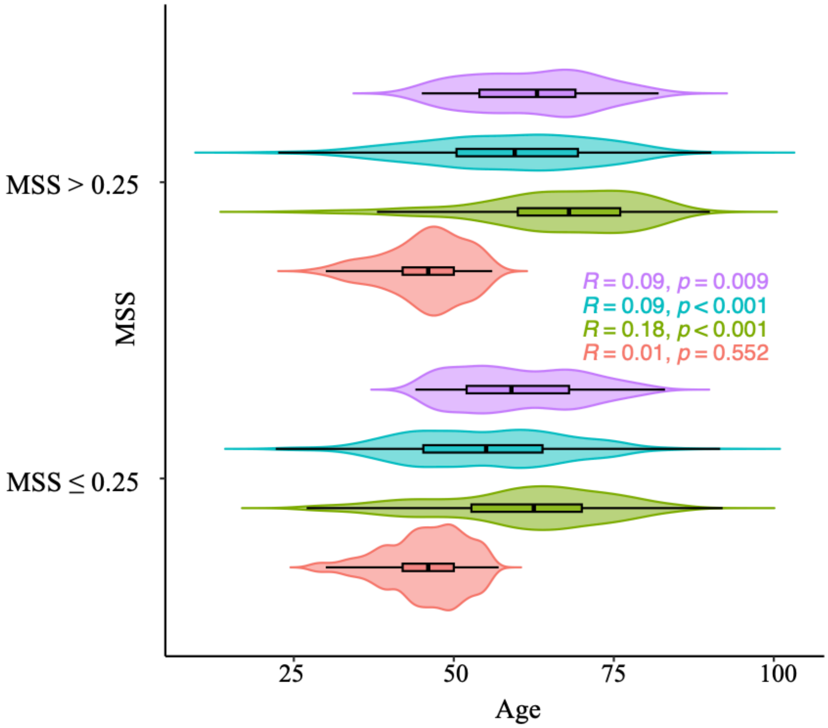

The figure shows the distributions of (a) heteroplasmic burden score (MHcount) based on rare variants, (b) heteroplasmic burden score (MH_{com}count) based on common variants, and (c) mitochondrial local constraint score sum (MSS) based on rare heteroplasmic variants across chronological age in each cohort (i.e., CARDIA (red), FHS (blue), JHS (green), MESA (purple)). For MSS, participants were divided into two groups with a threshold of 0.25, with the group "MSS > 0.25" representing participants harboring a higher proportion of deleterious heteroplasmic variants. In each violin plot, the box represents the median (vertical line in the box) and the 25% and 75% quantiles (box edges).

Figure S2. Associations and meta-analyses of mtDNA heteroplasmy burden with epigenetic aging metrics in sex-stratified analyses

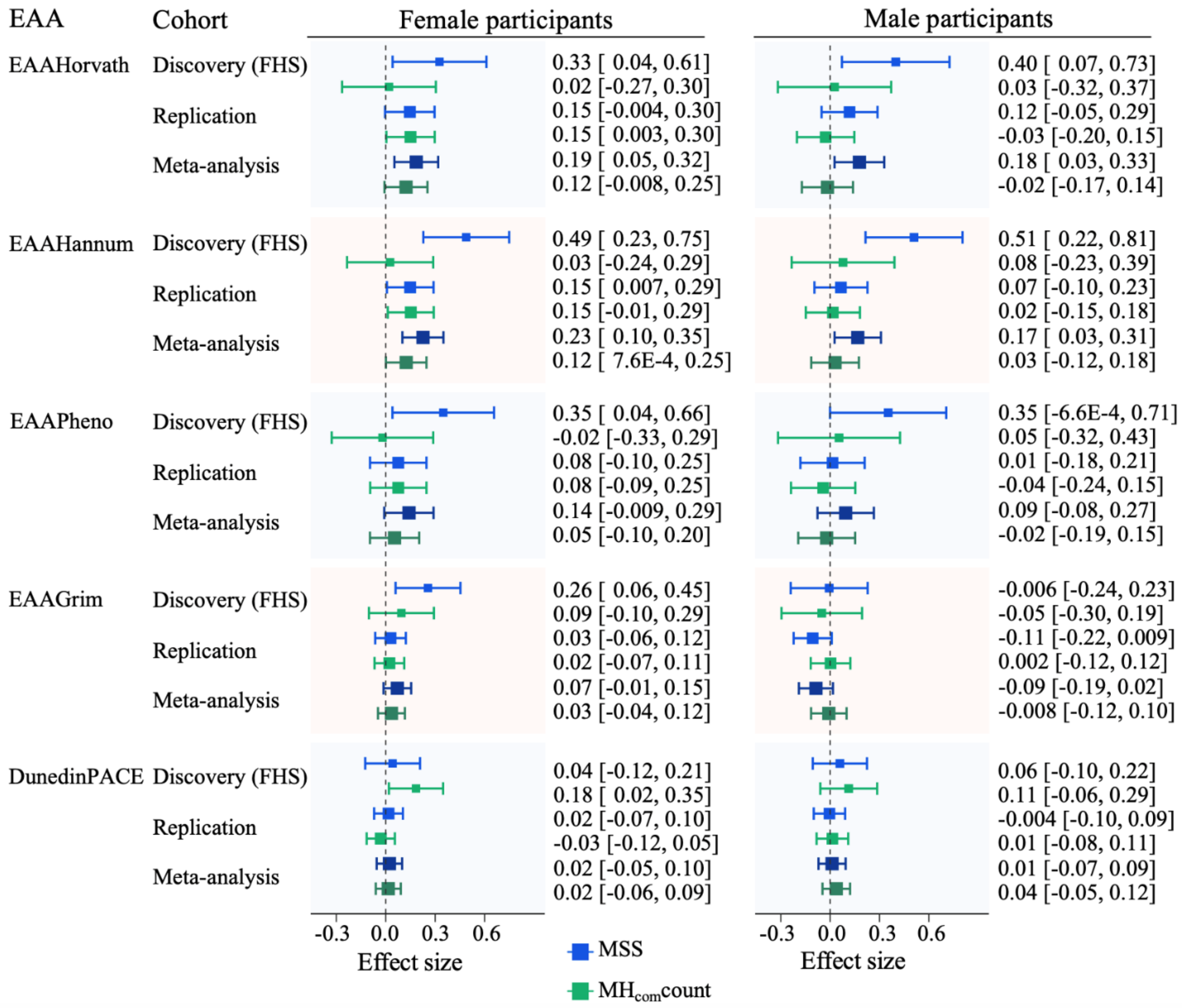

All association analyses were adjusted for chronological age, age square, smoking status, proportions of white blood cell compositions, and batch effects.
In meta-analysis, we utilized the inverse variance weighted fixed-effect meta-analysis to summarize association analyses across four cohorts. The beta coefficients represented the change in EAA with 1-SD higher level of MSS and MH_{com}count. The beta coefficients of DunedinPACE represented the change of 20-year DunedinPACE with one-SD higher level of MSS and MH_{com}count.
EAA, epigenetic age acceleration; MSS, mitochondrial local constraint score sum based on rare variants; MH_{com}count, burden score based on common variants.

Figure S3. Correlation between effect sizes from models with and without maternal structure adjustment in FHS

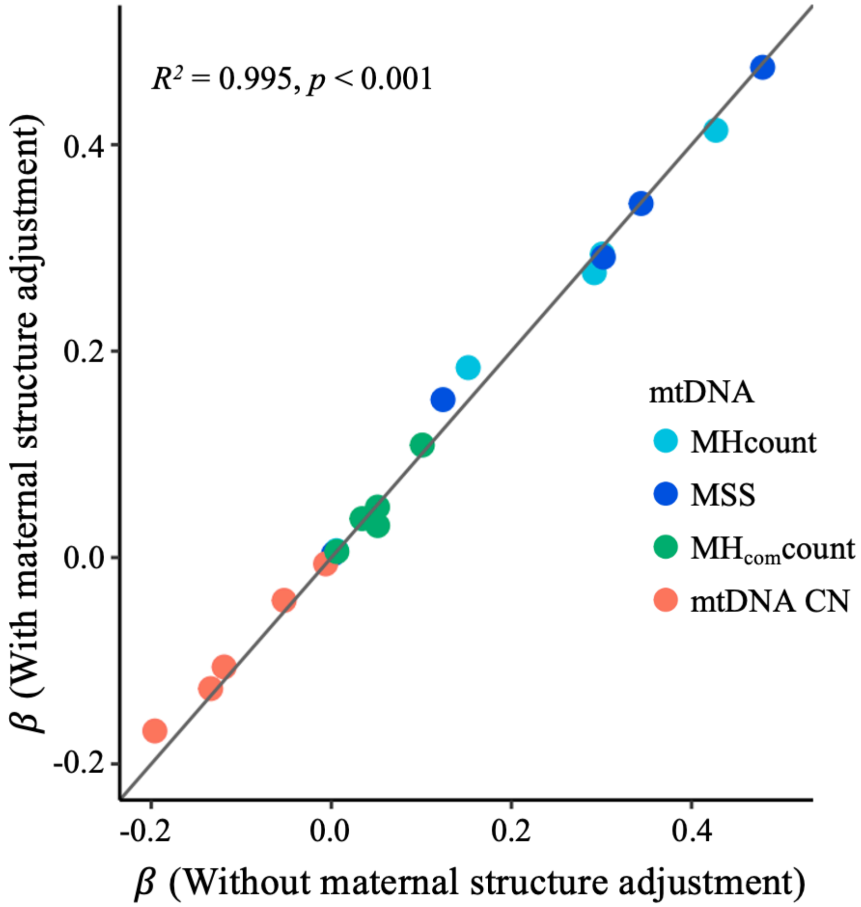

This figure indicated the correlation of effect sizes between the primary analysis and the sensitivity analysis with maternal structure adjustment in FHS. All association analyses were adjusted for chronological age, age square, sex, smoking status, proportions of white blood cell compositions, batch effects, and lab index with the same sample size (n = 1745). Sensitivity analyses were additionally adjusted for the maternal structure as the random effect. Effect sizes in all models represented the change of EAA with one-SD higher level of mtDNA metrics.

MHcount: mitochondrial heteroplasmic variant count based on rare variants; MSS: mitochondrial local constraint score sum based on rare variants; MH_{com}count: score based on mitochondrial common variants count; mtDNA CN: mitochondrial DNA copy number.

Figure S4. Correlation of effect sizes and significances from meta-analysis of MSS and mtDNA CN with EAAs between Black and White American participants

a.

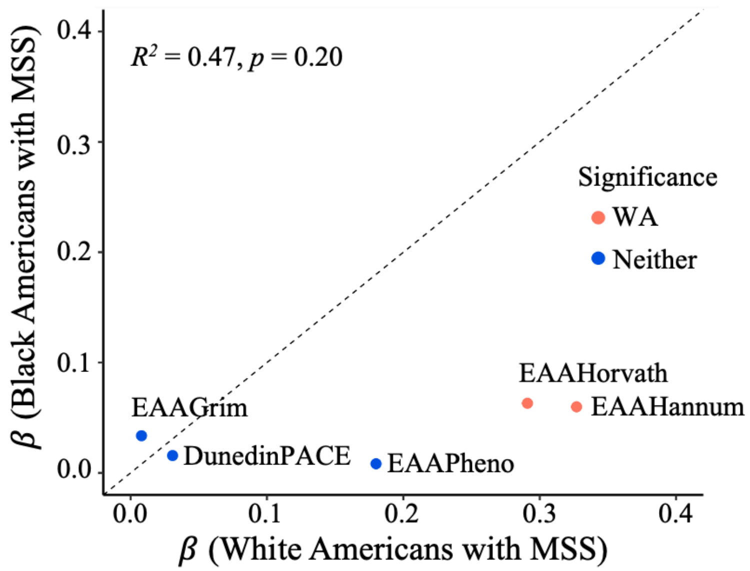

b.

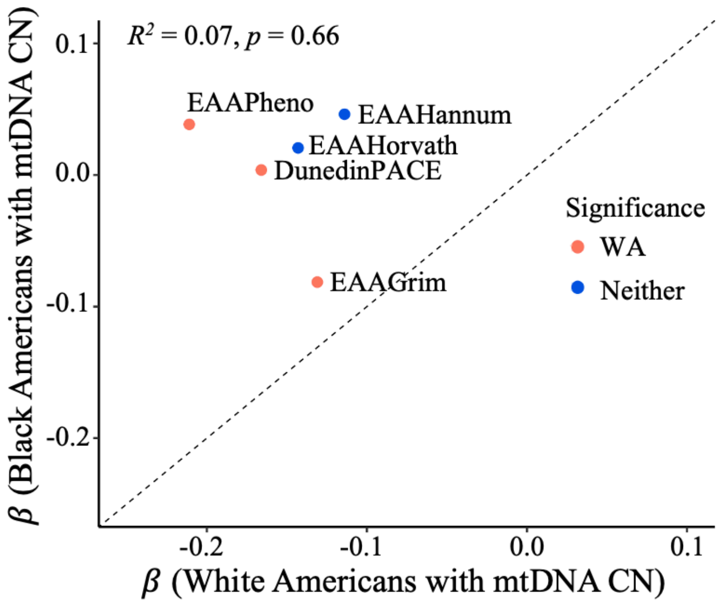

We conducted association analyses between mtDNA metrics (MSS and mtDNA CN) and EAAs in White Americans and Black Americans. We utilized the inverse-variance weighted fixed-effect meta-analysis to combine race/ethnicity-specific results across four cohorts (i.e., CARDIA, FHS, JHS, and MESA). The beta coefficients represented the change in EAA with one-SD higher level of MSS or mtDNA CN. The beta coefficients of DunedinPACE represented the change of 20-year DunedinPACE with 1-SD higher level of MSS or mtDNA CN.
MSS, mitochondrial local constraint score sum based on rare variants; mtDNA CN, mtDNA copy number.

Figure S5. Association analyses between MSS and DNA methylation probes of epigenetic aging metrics in FHS participants

a.

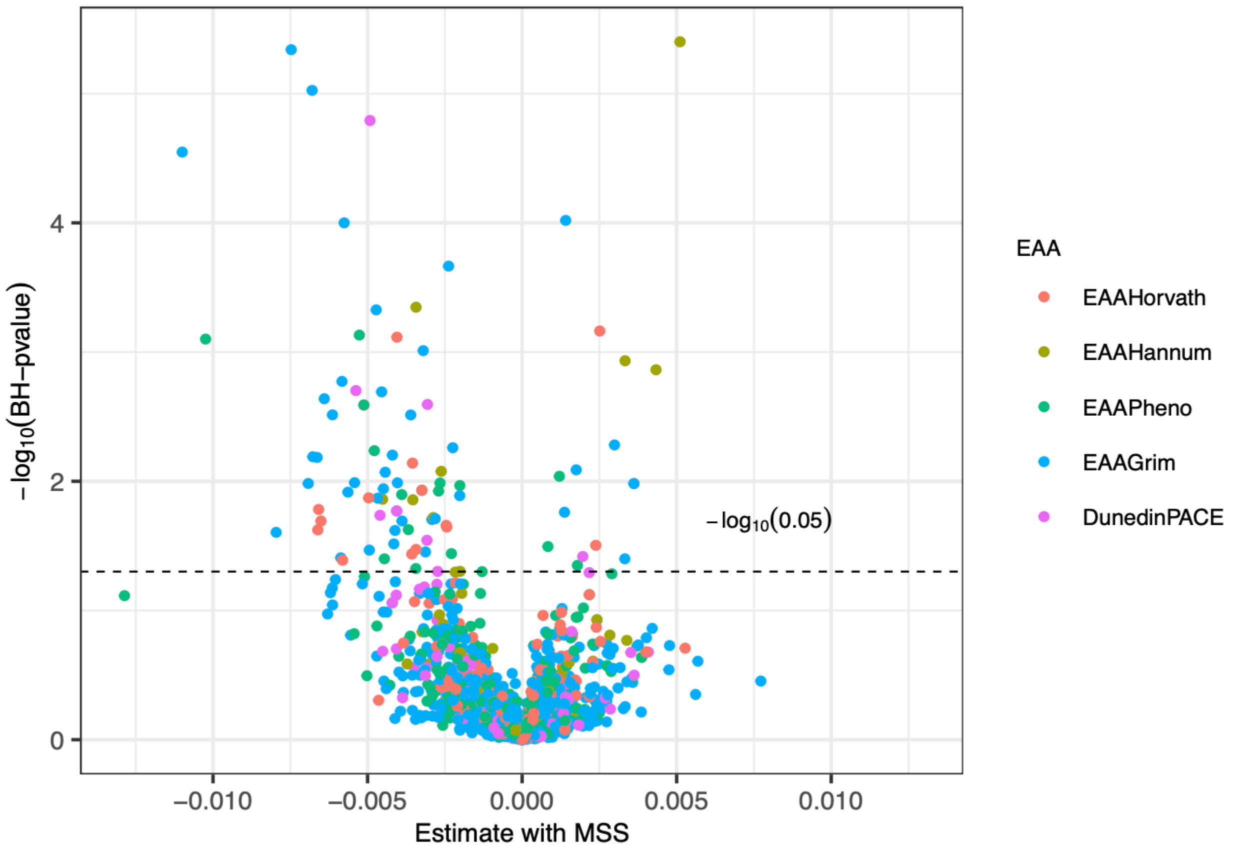

b.

	EAAHorvath	EAAHannum	EAAPheno	EAAGrim	DunedinPACE
MSS	15	10	16	40	8

(a) We conducted association analyses of 1950 distinct CpGs that were used to construct epigenetic age accelerations (EAAs) with mitochondrial burden score based on functions of rare heteroplasmic variants (MSS) in the FHS; (b) number of MSS-associated CpG probes (BH-adjusted p-value < 0.05).

We utilized 1950 unique CpG probes from DNA methylation age calculator and DunedinPACE calculation, then conducted association analysis of each CpG probe with MSS in the FHS, adjusting for chronological age, age squared, sex, smoking status, proportions of white blood cell compositions, batch effects, and the lab index. CpG probes were grouped into five colors (red: EAAHorvath, olive: EAAHannum, green: EAAPheno, blue: EAAGrim, purple: DunedinPACE).

MSS, mitochondrial local constraint score sum based on rare variants.

Figure S6. Associations and meta-analyses of mtDNA copy number with epigenetic aging metrics in sex-stratified analyses

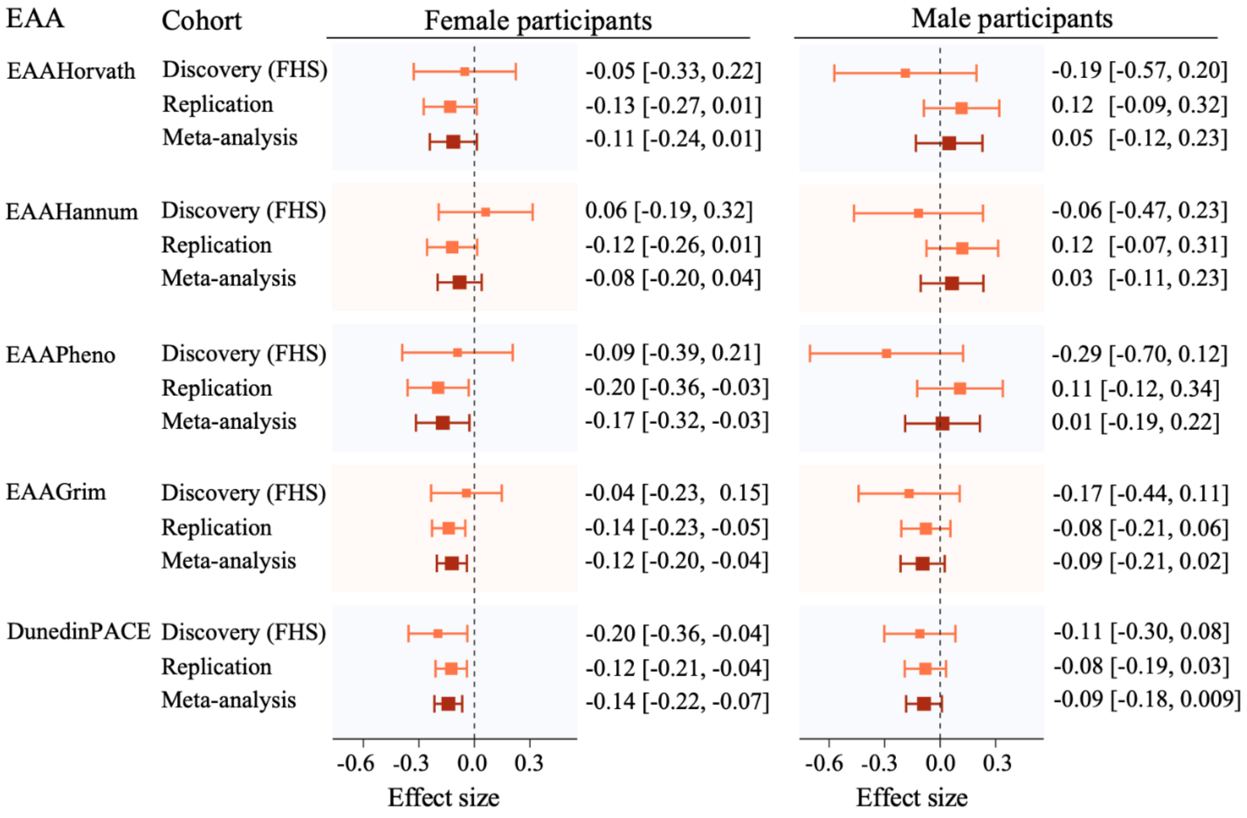

This figure indicated the comparison of associations between mtDNA copy number and EAA metrics between female and male participants. All association analyses were adjusted for chronological age, age squared, smoking status, proportions of white blood cell compositions, and batch effects.

In the meta-analysis, we utilized the inverse variance weighted fixed-effect meta-analysis to summarize association analyses across four cohorts. The beta coefficients represented the change in EAA with 1-SD higher level of mtDNA CN. The beta coefficients of DunedinPACE represented the change of 20-year DunedinPACE with one-SD higher level of mtDNA CN.

Figure S7. Mendelian randomization analysis: scatter plots of the causal relationship from mtDNA CN to EAAPheno and EAAGrim

a. Mendelian randomization from mtDNA CN to EAAPheno

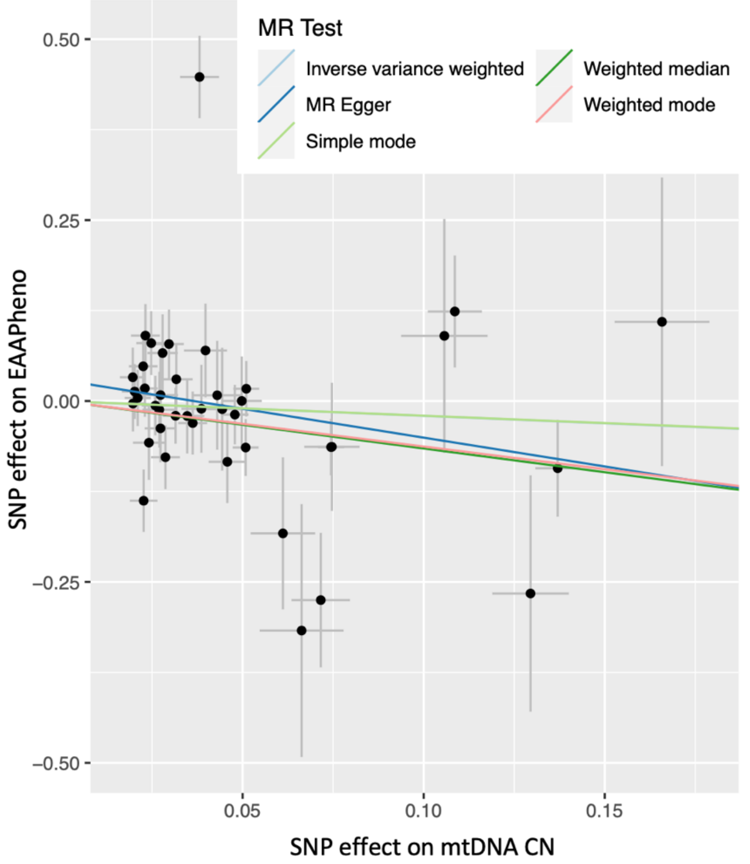

b. Mendelian randomization from mtDNA CN to EAAGrim

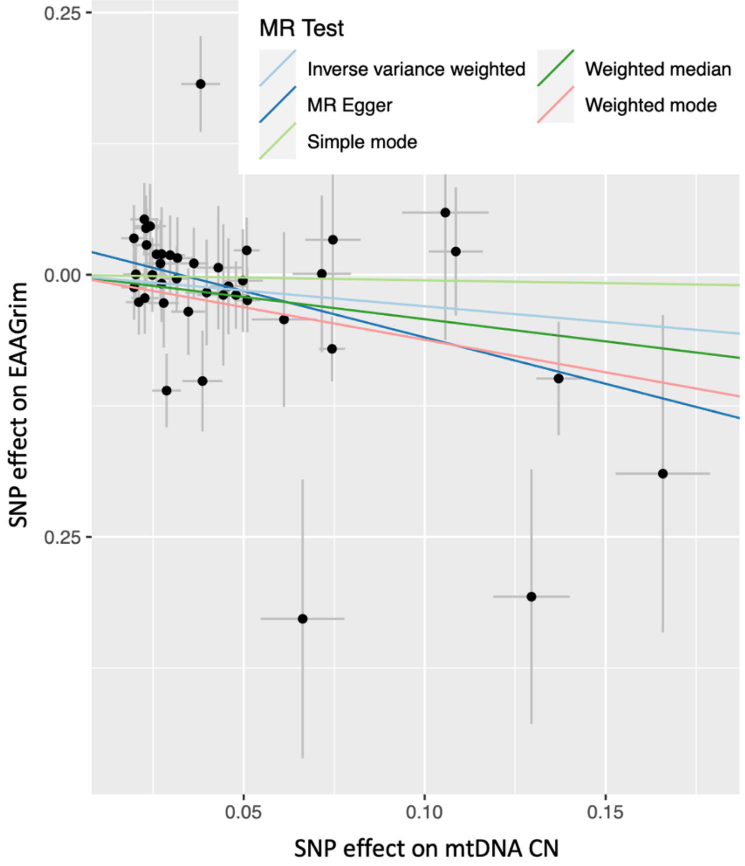

This figure showed the Mendelian randomization (MR) analysis from mtDNA CN to non-PC based (a) EAAPheno and (b) EAAGrim. Each dot in the figure represents one SNP. Inverse variance weighted method (light blue line) was used in our primary analysis. The MR analyses with MR-Egger (dark blue line) or weighted-median (dark green line) methods were sensitivity analyses. The MR-Egger method identified and adjusted for the potential pleiotropy effect. The weighted-median method was used to correct for the potential invalid instruments.

Figure S8. Mendelian randomization analysis: leave-one-out plot of the causal relationship from mtDNA CN to EAAPheno and EAAGrim with inverse-weighted variance method

a. Mendelian randomization from mtDNA CN to EAAPheno b. Mendelian randomization from mtDNA CN to EAAGrim

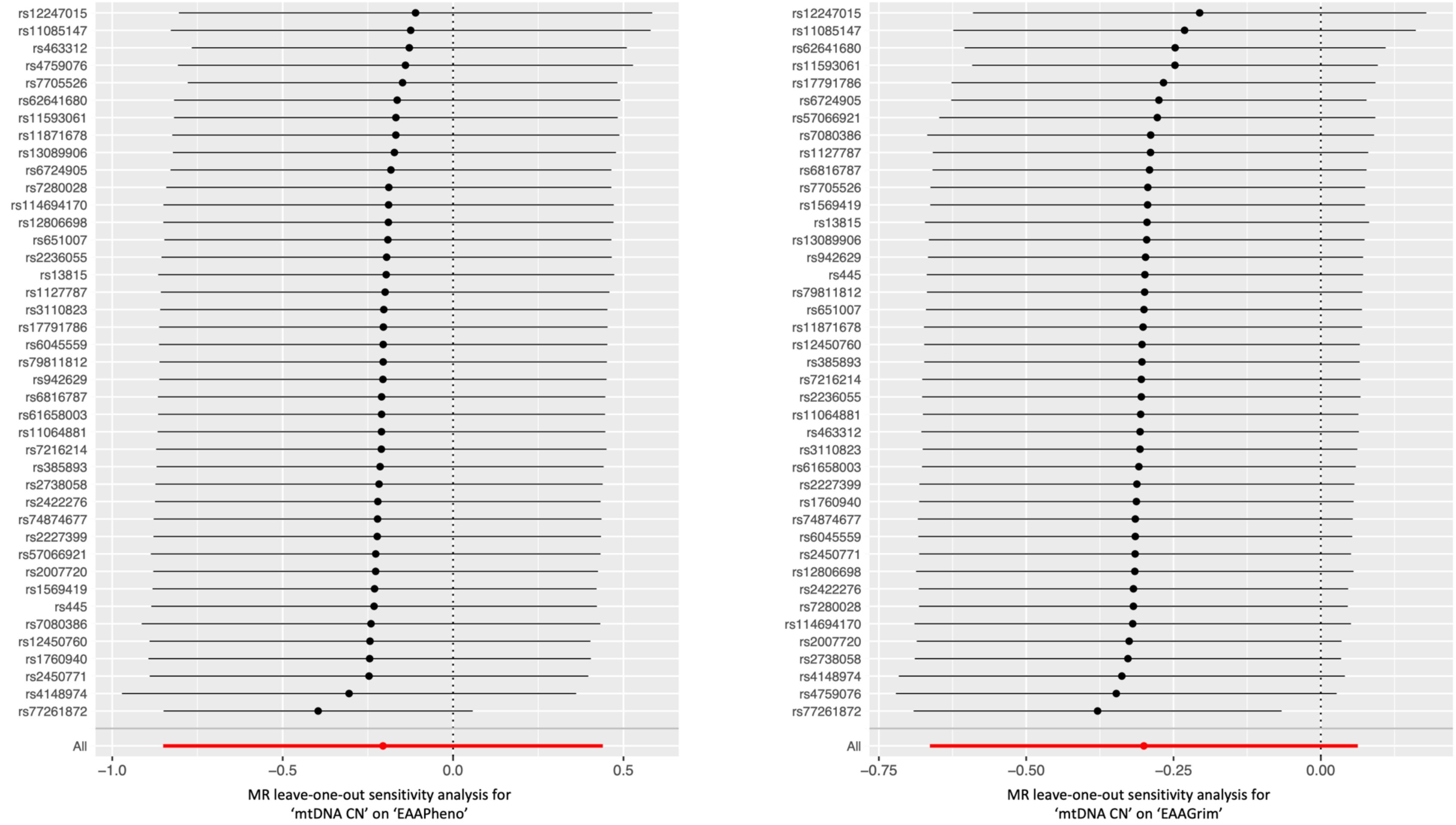

This figure represents the leave-one-out plot from mtDNA CN to non-PC based (a) EAAPheno and (b) EAAGrim in the Mendelian randomization (MR) analyses with inverse-weighted variance method. For each SNP, the MR analysis was performed after removing this SNP to assess if this SNP greatly influenced MR results.

Figure S9. Mendelian randomization analysis: leave-one-out plot of the causal relationship from mtDNA CN to EAAPheno and EAAGrim with MR-Egger method

a. Mendelian randomization from mtDNA CN to EAAPheno

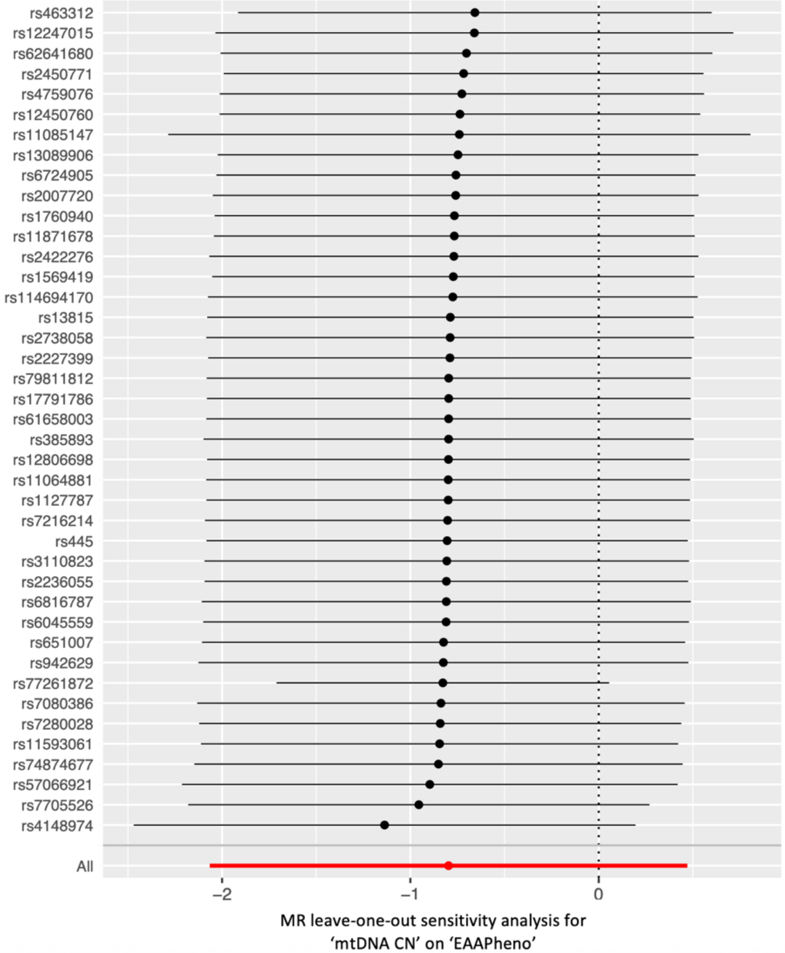

b. Mendelian randomization from mtDNA CN to EAAGrim

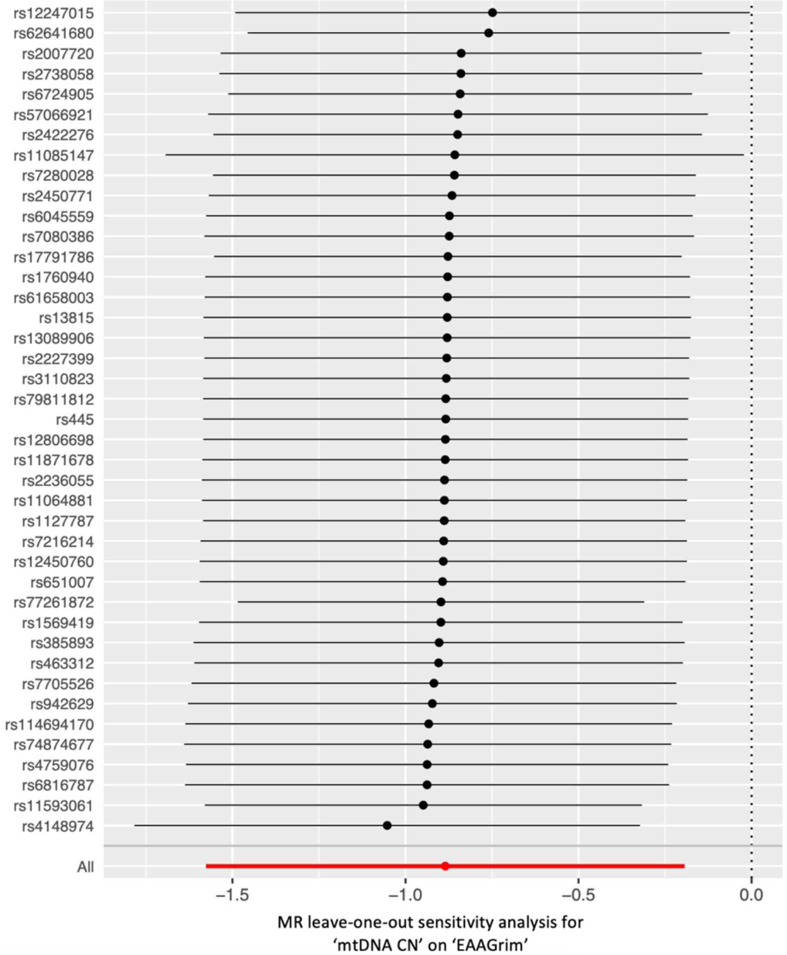

This figure represents the leave-one-out plot from mtDNA CN to non-PC based (a) EAAPheno and (b) EAAGrim in the Mendelian randomization (MR) analyses with MR-Egger method. For each SNP, the MR analysis was performed after removing this SNP to assess if this SNP greatly influenced MR results.

Figure S10. Mendelian randomization analysis: leave-one-out plot of the causal relationship from mtDNA CN to EAAPheno and EAAGrim with MR-median method

a. Mendelian randomization from mtDNA CN to EAAPheno

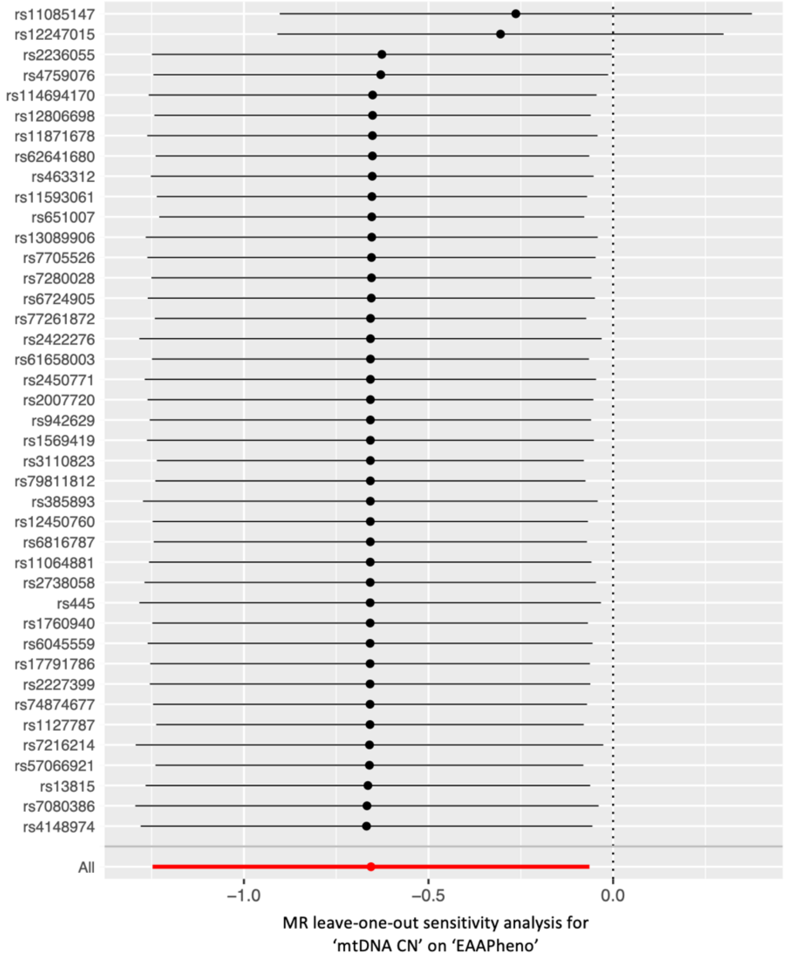

b. Mendelian randomization from mtDNA CN to EAAGrim

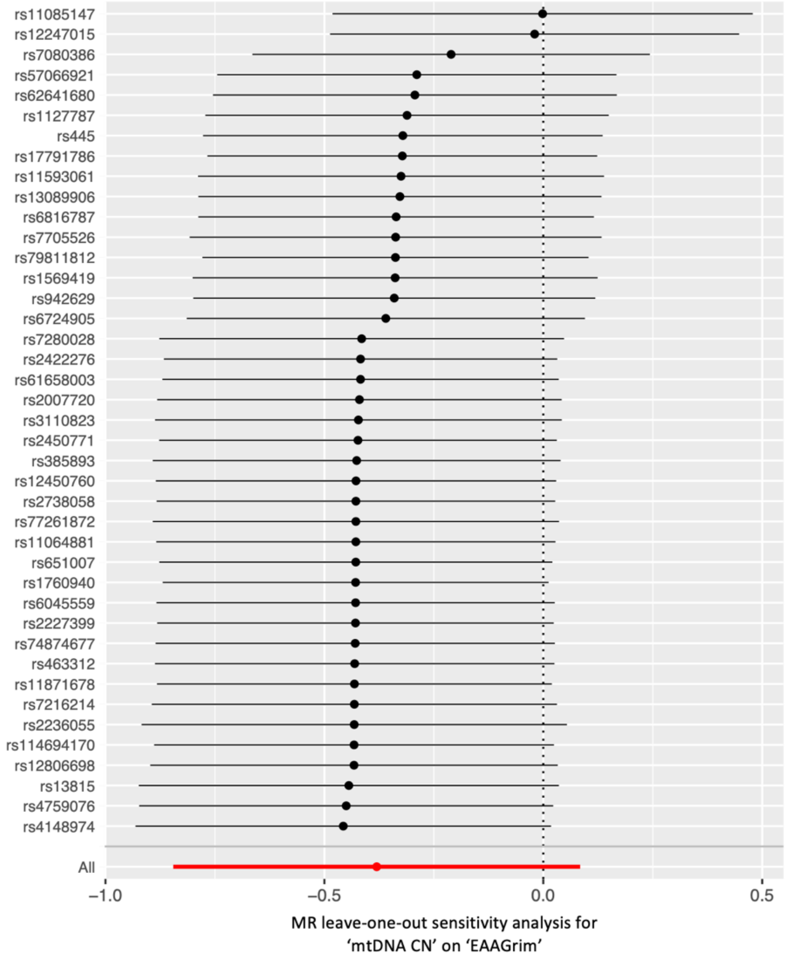

This figure represents the leave-one-out plot from mtDNA CN to non-PC based (a) EAAPheno and (b) EAAGrim in the Mendelian randomization (MR) analyses with MR-median method. For each SNP, the MR analysis was performed after removing this SNP to assess if this SNP greatly influenced MR results.

REFERENCES

Andersson C, Johnson AD, Benjamin EJ, Levy D & Vasan RS (2019) 70-year legacy of the Framingham Heart Study. *Nat Rev Cardiol* 16, 687–698.

Bild DE (2002) Multi-Ethnic Study of Atherosclerosis: Objectives and Design. *American Journal of Epidemiology* 156, 871–881.

Blaha MJ & DeFilippis AP (2021) Multi-Ethnic Study of Atherosclerosis (MESA). *Journal of the American College of Cardiology* 77, 3195–3216.

Chen Y, Lemire M, Choufani S, Butcher DT, Grafodatskaya D, Zanke BW, Gallinger S, Hudson TJ & Weksberg R (2013) Discovery of cross-reactive probes and polymorphic CpGs in the Illumina Infinium HumanMethylation450 microarray. *Epigenetics* 8, 203–209.

Dawber TR, Meadors GF & Moore FE (1951) Epidemiological Approaches to Heart Disease: The Framingham Study. *Am J Public Health Nations Health* 41, 279–286.

Feinleib M, Kannel WB, Garrison RJ, McNamara PM & Castelli WP (1975) The framingham offspring study. Design and preliminary data. *Preventive Medicine* 4, 518–525.

Kuan PF, Wang S, Zhou X & Chu H (2010) A statistical framework for Illumina DNA methylation arrays. *Bioinformatics* 26, 2849–2855.

Liu C, Marioni RE, Hedman ÅK, Pfeiffer L, Tsai P-C, Reynolds LM, Just AC, Duan Q, Boer CG, Tanaka T, Elks CE, Aslibekyan S, Brody JA, Kühnel B, Herder C, Almli LM, Zhi D, Wang Y, Huan T, Yao C, Mendelson MM, Joehanes R, Liang L, Love S-A, Guan W, Shah S, McRae AF, Kretschmer A, Prokisch H, Strauch K, Peters A, Visscher PM, Wray NR, Guo X, Wiggins KL, Smith AK, Binder EB, Ressler KJ, Irvin MR, Absher DM, Hernandez D, Ferrucci L, Bandinelli S, Lohman K, Ding J, Trevisi L, Gustafsson S, Sandling JH, Stolk L, Uitterlinden AG, Yet I, Castillo-Fernandez JE, Spector TD, Schwartz JD, Vokonas P, Lind L, Li Y, Fornage M, Arnett DK, Wareham NJ, Sotoodehnia N, Ong KK, van Meurs JBJ, Conneely KN, Baccarelli AA, Deary IJ, Bell JT, North KE, Liu Y, Waldenberger M, London SJ, Ingelsson E & Levy D (2018) A DNA methylation biomarker of alcohol consumption. *Mol Psychiatry* 23, 422–433.

Lloyd-Jones DM, Lewis CE, Schreiner PJ, Shikany JM, Sidney S & Reis JP (2021) The Coronary Artery Risk Development In Young Adults (CARDIA) Study. *Journal of the American College of Cardiology* 78, 260–277.

Sempos CT, Bild DE & Manolio TA (1999) Overview of the Jackson Heart Study: a study of cardiovascular diseases in African American men and women. *Am J Med Sci* 317, 142–146.

Splansky GL, Corey D, Yang Q, Atwood LD, Cupples LA, Benjamin EJ, D'Agostino RB, Fox CS, Larson MG, Murabito JM, O'Donnell CJ, Vasan RS, Wolf PA & Levy D (2007) The Third Generation Cohort of the National Heart, Lung, and Blood Institute's Framingham Heart Study: Design, Recruitment, and Initial Examination. *American Journal of Epidemiology* 165, 1328–1335.

Taguchi Y -h. & Oono Y (2005) Relational patterns of gene expression via non-metric multidimensional scaling analysis. *Bioinformatics* 21, 730–740.

Wilson JG, Rotimi CN, Ekunwe L, Royal CDM, Crump ME, Wyatt SB, Steffes MW, Adeyemo A, Zhou J, Taylor HA & Jaquish C (2005) Study design for genetic analysis in the Jackson Heart Study. *Ethn Dis* 15, S6-30–37.
